# Expression and function of the *cdgD* gene, encoding a CHASE–PAS-DGC-EAL domain protein, in *Azospirillum brasilense*

**DOI:** 10.1038/s41598-020-80125-3

**Published:** 2021-01-12

**Authors:** José Francisco Cruz-Pérez, Roxana Lara-Oueilhe, Cynthia Marcos-Jiménez, Ricardo Cuatlayotl-Olarte, María Luisa Xiqui-Vázquez, Sandra Raquel Reyes-Carmona, Beatriz Eugenia Baca, Alberto Ramírez-Mata

**Affiliations:** Laboratorio de La Interacción Bacteria-Planta, Centro de Investigaciones en Ciencias Microbiológicas, Benemérita Universidad Autónoma de Puebla, Avenida San Claudio S/N, Puebla Pue, Mexico

**Keywords:** Microbiology, Molecular biology

## Abstract

The plant growth-promoting bacterium *Azospirillum brasilense* contains several genes encoding proteins involved in the biosynthesis and degradation of the second messenger cyclic-di-GMP, which may control key bacterial functions, such as biofilm formation and motility. Here, we analysed the function and expression of the *cdgD* gene, encoding a multidomain protein that includes GGDEF-EAL domains and CHASE and PAS domains. An insertional *cdgD* gene mutant was constructed, and analysis of biofilm and extracellular polymeric substance production, as well as the motility phenotype indicated that *cdgD* encoded a functional diguanylate protein. These results were correlated with a reduced overall cellular concentration of cyclic-di-GMP in the mutant over 48 h compared with that observed in the wild-type strain, which was recovered in the complemented strain. In addition, *cdgD* gene expression was measured in cells growing under planktonic or biofilm conditions, and differential expression was observed when KNO_3_ or NH_4_Cl was added to the minimal medium as a nitrogen source. The transcriptional fusion of the *cdgD* promoter with the gene encoding the autofluorescent mCherry protein indicated that the *cdgD* gene was expressed both under abiotic conditions and in association with wheat roots. Reduced colonization of wheat roots was observed for the mutant compared with the wild-type strain grown in the same soil conditions. The *Azospirillum*-plant association begins with the motility of the bacterium towards the plant rhizosphere followed by the adsorption and adherence of these bacteria to plant roots. Therefore, it is important to study the genes that contribute to this initial interaction of the bacterium with its host plant.

## Introduction

*Azospirillum brasilense* is a species that has been employed as an inoculant worldwide because of its beneficial effects on host plants via multiple mechanisms, which are proposed to be responsible for the improved nutritional status of plants. These mechanisms include both direct effects on plant metabolism by providing nutrients (iron, phosphorus and nitrogen) and synthesizing molecules with phytohormone activity (auxins, cytokinins, gibberellins, and nitric oxide) and indirect effects such as antagonism against phytopathogens and increased resistance to pathogens, which define the mode of action of *Azospirillum* in plant development according to the additive hypothesis^1^. Therefore, this bacterium must contain an array of genes that participate in the control of the successful colonization of plants through such processes as chemotaxis and biofilm formation, which are the initial steps leading to the beneficial effects observed in plants of agronomic interest^2,3^.

Investigations into the role of the cyclic dimeric guanosine monophosphate (c-di-GMP) second messenger signaling system have shown that the predominant bacterial trait controlled by this system is the transition from a motile to a sessile lifestyle^4,5^ and these studies have indicated that the accumulation of c-di-GMP promotes biofilm formation via the biosynthesis of biofilm matrix components, adhesins, and exopolysaccharides^6–8^, while a decrease in c-di-GMP promotes motility, inhibiting this process^9,10^. The enzymes catalyzing c-di-GMP formation are diguanylate cyclases (DGCs), while those degrading c-di-GMP are phosphodiesterases (PDEs), which are identified by their characteristic GGDEF and EAL domains, respectively^11^. In bacteria, genes with translational products that include both domains (GGDEF and EAL) are referred to as hybrid proteins^11^.

Previous work has shown that *A. brasilense* Sp245 contains 35 genes that may encode proteins involved in the metabolism of c-di-GMP^12^. We identified 20 genes encoding presumptive proteins harboring only a GGDEF domain, five harboring only an EAL domain, and ten hybrid proteins with both domains. In addition, almost all of these proteins contained distinct N-terminal sensing and signaling domains, suggesting that their activities in cyclic-di-GMP turnover respond posttranslationally to various intra- and extracellular signals^7,11,12^.

Despite extensive studies conducted on other rhizosphere-colonizing bacteria to determine the genes responsible for the biosynthesis and degradation of c-di-GMP and their roles in plant colonization^7,13–15^, this information has not been obtained to date in *A. brasilense,* which is an important soil bacterium that is commonly used as an inoculant that promotes beneficial effects in a variety of plants. Thus, the aim of this work was to characterize the *cdgD* gene*,* which encodes a DGC-EAL hybrid protein referred to as CdgD. We showed that this gene is involved in motility and biofilm formation and demonstrated its expression both under abiotic conditions and in association with wheat roots, suggesting that it plays a prominent role in the interaction with the host plant.

## Materials and methods

### Strains, plasmids, primers and growth conditions

The strains, plasmids and primers used in this work are listed in Supplementary Tables S1 and S2. The *Azospirillum brasilense* Sp245 strain was grown at 30 °C in K-malate, Luria–Bertani modified (LB*) or Congo red (CR) solid medium as previously described^16^. The *A. brasilense* 12-A mutant, *A. brasilense* FPDm1, and *A. brasilense* T7mCh strains were grown in selective K-malate, LB* or CR solid medium supplemented with 50 μg/mL kanamycin (Km). *A. brasilense* C-56A was grown in CR medium supplemented with 20 µg/mL tetracycline (Tc), and *A. brasilense* derivative strains tagged with the mCherry protein harboring the pMP2449-5 plasmid were grown in CR supplemented with 30 μg/mL gentamicin (Gm). The *Escherichia coli* DH5α and S17.1 strains were grown at 37 °C in liquid LB medium, at pH 7.0, to which solid agar (1.5% agar, w/v) was added to produce solid medium. The selective media utilized for the *E. coli* strains were supplemented with ampicillin (Ap) at 100 μg/mL; Tc at 10 μg/mL; chloramphenicol (Cm) at 25 μg/mL; Gm at 15 μg/mL; or Km at 30 μg/mL. The solid mating medium used for conjugation was produced with LB* medium, and transconjugant selection was performed on solid K-malate medium supplemented with appropriate antibiotics as previously described^16^.

### Construction and selection of the *A. brasilense* 12-A null mutant, *A. brasilense* C-56A complemented mutant, and *A. brasilense* C-40A control strains

The *A. brasilense cdgD::*Km^R^ null mutant, referred to as the 12-A strain, was constructed as described previously^16^. Initially, the pAB*cdgD* suicide plasmid was produced by molecular cloning following the molecular biology protocols described by Green and Sambrook^17^. Briefly, a fragment containing the *cdgD* gene was obtained by PCR. The primers used for this purpose were 4571RC-F and 4571DC-R (Supplementary Table S2), and amplification was performed with High-fidelity Platinum *Taq* DNA polymerase (Invitrogen Thermo Fisher Scientific Life Technologies, Carlsbad, CA, USA). The fragment was cloned into the pCR 2.1 TOPO vector (Thermo Fisher Scientific Life Technologies) to yield the pCR*cdgD* plasmid. The pCR*cdgD* plasmid was digested with the restriction enzyme *EcoR*I, and the fragment was cloned into pSUP202, digested with the same restriction enzyme^18^, to yield pAB*cdgD*. The *km*^*R*^ gene was released from the pBSL98 plasmid with the restriction enzyme *Sma*I^19^ and inserted into pAB*cdgD*, which had been digested with the same restriction enzyme; this construct was named pAB*cdgD::km*^R^. The obtained plasmids were transformed into *E*. *coli* S17.1 cells^18^. For conjugation assays, *A. brasilense* cells were grown on LB* solid medium^16^. Transconjugants were selected in K-malate medium supplemented with Km at 50 μg/mL. The obtained mutant was verified by PCR and sequencing, which showed that the ORF was disrupted. The *A. brasilense* C-56A complemented mutant was generated from a construct of the *cdgD* gene with its native region promoter obtained with the primers F-ORF210 (*Hind*III) and R-ORF-210 (*Xho*I), containing the restriction enzyme sequences indicated for direct cloning in the pVK100 broad host plasmid^20^, which was digested with the same restriction enzymes to yield the construct pVK*pcdgD*. Next, this construct was transformed into *E*. *coli* DH5α cells and subsequently to the *E*. *coli* S17.1 (pVK*pcdgD*) donor. This plasmid was then transferred by conjugation to *A. brasilense* 12-A. A control strain carrying the empty plasmid was also constructed to generate the *A. brasilense* C-40A strain. All strains were verified by PCR and sequencing using the described corresponding primers (Supplementary Table S2).

### Construction of the *A. brasilense* FPDm1 chromosomal transcriptional fusion for abiotic and plant assays

The pAZBR-T7mCh plasmid was constructed from the pJMS-Km^R^ suicide vector^21^ as follows. The CMP-*Pst*IF/CMP-*Kpn*IR primers (Supplementary Table S2) were used to amplify a 5′ fragment of 700 bp that included the ORF (GenBank accession number CCC96879.1, encoding a membrane protein of unknown function), which was then cloned into pGEM-TEasy (Promega, Madison, WI, USA) to generate the pAB79 plasmid. Subsequently, the MTF-*Spe*IF/MTF-*Nco*IR primers (Supplementary Table S2) were used to amplify a 3′ fragment of 900 bp that include the ORF (GenBank accession number CCC96880.1, encoding a putative SAM-dependent methyltransferase), which was further cloned into pGEM-TEasy to yield the pAB80 plasmid. Both fragments were excised from the plasmids with the corresponding restriction enzymes and cloned into pJMS-Km^R^ to obtain a suicide plasmid with the *mCherry* gene without a promoter. Subsequently, the amplicon of the terminator of the T7 sequence (T7 iorA, Supplementary Table S2) was introduced between the *mCherry* gene and the CCC96879.1 gene to guarantee the transcription termination of the ORF encoded by this gene. This plasmid was designated pAZBR-T7mCh (Supplementary Fig. S1). In this plasmid, a fragment 456 bp upstream of the *cdgD* gene, including its promoter, which had been previously amplified with the primers P-*cdgD*-F/(*Sna*B1) and P-*cdgD*-R/(*Xho*I) (Supplementary Table S2), was cloned into the 5´ region of the *mCherry* gene, which was previously cloned into pGEM-TEasy to yield the pGEM-*pcdgD* plasmid. Both the pAZBR-T7mCh and pGEM-*pcdgD* plasmids were digested with *Xho*I and *Sna*B1, followed by cloning to obtain the pAZBR*pcdgD*T7mCh plasmid, which was verified by PCR and sequencing. The pAZBR-T7mCh and pAZBR*pcdgD*T7mCh constructs were subsequently transformed into the mobilizing *E. coli* S17.1 strain, and the resultant strains were then transferred by conjugation into the *A. brasilense* Sp245 strain (cloned into a neutral locus of the *A. brasilense* chromosomal region) to obtain the control and tagged mCherry strains, respectively. These last two strains were used in further experiments to determine the expression of the *cdgD* gene under culture conditions and in wheat plants (Supplementary Table S1 and Fig. S1).

### Application of bioinformatics tools for the analysis and modeling of the CdgD protein

The CdgD protein sequence from *A. brasilense* Sp245 was retrieved from the NCBI database (https://www.ncbi.nlm.nih.gov/protein; accession number; AZOBR_100210). The Rapid Annotation using Subsystem Technology (RAST, http://RAST.nmpdr.org) server was employed for the initial analysis. Protein domain predictions were made using SMART^22^ (http://smart.embl-heidelberg.de), HMMER^23^ and PROSITE^24^. To predict and identify transmembrane regions, the TMHMM^25^ and TMpred tools were used^26^. The visualization of the transmembrane helices was performed with the HeliQuest helical wheel-drawing program^27^. Secondary and three-dimensional structure predictions were performed using the Phyre2 and I-Tasser programs to detect the domain organization and to identify a suitable template fold for the CHASE and PAS-GGDEF-EAL domains^28^. Three-dimensional models of the CHASE (residues 64–327) and PAS-GGDEF-EAL (residues 378–946) domains were obtained using the Phyre2 and I-Tasser packages and analysed with Chimera software^29^ using the following crystal structure as a structural templates: the CHASE domain of the PcrK histidine kinase of *Xanthomonas campestris* pv *campestris* (Protein Data Bank, PDB Code: 6K62)^30^, histidine kinase 4 (AHK4) from *Arabidopsis thaliana* (PDB Code: 3T4J)^31^, and the PAS-GGDEF-EAL domains of the RbdA protein from *Pseudomonas aeruginosa* (PDB Code: 5XGB)^32^, as reference models.

### Biofilm assays

Overnight cultures in LB* were subcultured in fresh NFB* supplemented with KNO_3_ or NH_4_Cl as a nitrogen source to an OD at 590 nm (OD 590) of 0.01 and grown with shaking at 30 °C until they reached an OD 590 of 1.2–1.4, as described previously^33^. The cultures were diluted 1:100, and a 3 mL aliquot was inoculated into each of four wells per strain in a 24-well plate. The plates were incubated in a humidified chamber at 30 °C for 72 h. Then, the adherent biomass was stained via immersion for 30 min in 0.5 mL of a 0.5% (w/v) crystal violet solution. The adsorbed crystal violet was solubilized by immersion in 2 mL of 33% acetic acid, and the absorbance of this solution was determined at 595 nm in a multidetection microplate reader (EON-Biotek Spectrophotometer Biotek Instrument). The biomass of each sample was determined by total protein determination with the Bradford reagent assay (Sigma-Aldrich Chemical, St. Louis, Missouri, USA). The data are presented as ratios normalized to the mg/protein values obtained within each experiment. The wild-type, mutant, and complemented mutant were evaluated in three independent experiments, each of which included three technical replicates.

### Motility assays

The motility assays were performed essentially as described previously^21^ in media containing 0.25% agar and K-malate, K-fumarate and K-proline as a source of carbon at a 10 mM concentration. The wild-type, mutant, and complemented mutant were evaluated by measuring their motility in cm in three independent experiments, each of which included three technical replicates.

### Determination of extracellular polymeric substance (EPS) production

EPS quantification was performed as previously described^21^. Briefly, the *A. brasilense* WT strain, its isogenic mutant, and the complemented strain were grown in NFB* medium supplemented with KNO_3_ or NH_4_Cl as a nitrogen source to an OD at 590 nm of 0.01 and subsequently grown with shaking at 30 °C until they reached an OD at 590 nm of 1.2–1.4. These cultures were incubated at 30 °C under static conditions for 3 days. The cells (2 mL) were harvested by centrifugation at 10,000 rpm (Biofuge-Heraeus, Thermo Fisher Scientific) for 3 min and resuspended in 1 mL of NFB* medium, to which a 0.005% (w/v) CR colorant solution (Sigma-Aldrich, Chemical) was added to achieve a 40 µg/mL concentration. The cells were incubated with shaking (200 rpm) for 2 h. Next, the samples were pelleted by centrifugation at 10,000 rpm. The amount of CR remaining in the supernatant was determined by measuring the OD 490 nm of the solution and comparing the result with the calibration curve of CR (concentration from 10 to 300 µg/mL) to obtain the µg CR. The protein concentration was determined via the Bradford protein assay. CR binding was expressed as µg CR/mg protein. Each culture was evaluated by measuring the EPS content in three independent experiments, each of which included three technical replicates.

### Determination of cyclic-di-GMP levels using a riboswitch biosensor

*Azospirillum brasilense* strains harboring the pFY4535 plasmid containing the c-di-GMP biosensor^34^ were constructed via the conjugation of *E. coli* S17.1 (pFY4535) to *A. brasilense* Sp245, *A. brasilense* 12-A, *A. brasilense* C-56A and *A. brasilense* C-40A as described previously^16^. Next, the reporter strains were streaked onto a Congo red agar plate containing 30 μg/ml gentamicin and incubated for 48 h at 30 °C. Two colonies from each plate were inoculated into NFB* + KNO_3_ broth containing 30 μg/mL Gm and incubated for 24, 48, and 72 h at 30 °C with shaking at 120 rpm. The *Azospirillum* cells were mounted on a coverslip and sealed with a 1% agar plug. Images were acquired using a Nikon TE2000U microscope equipped with a 100× objective (oil immersion objective). For the AmCyan fluorophore, excitation was performed at 457 nm, and the emission spectrum was obtained at 520 nm (green). For the TurboRFP fluorophore, the excitation spectrum was obtained at 553 nm, and emission was measured at 574 nm (red). The images were edited and analysed with Nikon NIS Elements and ImageJ software^35^, respectively. Relative fluorescence intensity (RFI) values were calculated by dividing the intensity value of TurboRFP by that of AmCyan.

### RNA extraction and quantitative RT-qPCR

*Azospirillum brasilense* Sp245 was grown in NFB* medium supplemented with KNO_3_ or NH_4_Cl as a nitrogen source^33^ under planktonic or biofilm conditions for 10, 18, 24 or 48 h as described above. After the cells from 6 mL of the culture were pelleted and washed with TE (10 mM Tris HCl, 1 mM EDTA) buffer, pH 8.0, the obtained pellets were stored at − 70 °C until use. Total RNA was extracted from the pellets, resuspended in 550 µL of TE buffer plus 200 µg/mL (w/v) lysozyme and lysed by sonication at 30% for 5 s (Hielscher UP50H—Compact Lab Homogenizer, 12623 Berlin Germany). The cell suspension was maintained at 37 °C with shaking for 35 min, and 50 µL of 10% SDS (w/v) plus 400 µL of cetyl trimethyl ammonium bromide (CTAB 2% w/v in NaCl 2 M; CTAB, Sigma-Aldrich) was added. After mixing, the suspension was centrifuged at 10,000 rpm for 10 min. The supernatant was treated by mixing with 150 µL of phenol-Tris–HCl, pH 8.0, and RNA was extracted four times with 400 µL of chloroform/isoamyl alcohol (v/v 24:1). After that step, the RNA was precipitated with ethanol and centrifuged at 10,000 rpm for 10 min. The RNA was resuspended in 50 µL of water treated with diethylpyrocarbonate (DEPC, Sigma-Aldrich) at a concentration of 0.1% (w/v) and stored at − 70 °C until use. The concentration and quality of the RNA were determined using an EON-BioTeK spectrophotometer (BioTek Instruments Inc.). RNA integrity was confirmed by electrophoresis in 0.8% (w/v) agarose gels. Complementary DNA (cDNA) synthesis by reverse transcription (RT-PCR) was performed using the Maxima First Strand cDNA Synthesis system (Thermo Fisher Scientific) from 1 µg of extracted RNA. For RT-qPCR analysis, three liquid culture biological replicates were performed for each gene (*cdgD* and *glyA*). Specific RT-qPCR primers (qRT-*cdgD*F, qRT-*cdgD*R, and glyAF, glyAR^36^); were used to amplify the reference and target genes. The reactions were carried out in triplicate using SYBR Green High ROX master mix (Thermo Fisher Scientific, USA). The thermocycling conditions were as follows: 95 °C for 3 min, 40 repetitions of 95 °C for 30 s and 60.5 °C for 30 s, followed immediately by a melting curve, in a StepOnePlus thermocycler (Applied Biosystems, Foster City, California, USA). Gene expression was normalized to the level of the endogenous housekeeping gene *glyA* and to the wild-type RNA reference sample following the 2^−ΔΔC^_T_ method. The standard curve was constructed (in duplicate) using chromosomal DNA. Melting curve analysis was used to confirm the production of a specific single product by each primer pair.

### Plant growth conditions of the *A. brasilense* strains, inoculation, and visualization of colonization by confocal laser scanning microscopy (CLSM)

The plant experiments were performed essentially as previously described^37^ using seeds of the wheat (*Triticum aestivum*) variety ‘Nana’ and soil obtained from the Campo Agrícola Experimental del Valle de México (INIFAP). Briefly, the seeds were surface-sterilized with 1% (v/v) sodium hypochlorite with agitation (60 rpm) for 30 min after rinsing with sterile water and were then immersed in 40 mL of a solution containing 150 μg/mL cycloheximide, 250 μg/mL streptomycin, 20 μg/mL Tc, and 150 μg/mL fluconazole, with agitation at 60 rpm for 10 min. At the end of the disinfection process, the seeds were rinsed five times with demineralized sterile water for 10 min. Regarding seed inoculation, the *A. brasilense* Sp245, *A. brasilense* 12-A, *A. brasilense* C-40A and *A. brasilense* C-56A strains harboring the pMP2449-5 plasmid were grown on K-malate medium overnight at 30 °C with shaking (150 rpm). The bacterial cells were resuspended in K-malate medium at a final density of 10^9^ colony-forming units (CFU)/mL (OD 600 = 1.2). The bacterial suspensions were diluted 100-fold in sterilized NFB* medium to a final density of approximately 5 × 10^6^–5 × 10^7^ CFU/mL. The inoculated seedlings were maintained in tubes with 15 mL of Hoagland hydroponic solution supplemented with NH_4_NO_3_ (4 mM) in a growth chamber for 7 days under a day–night cycle of 14 h light at 24 °C and 10 h darkness at 16 °C with humidity of 80% as previously described^37^. At 7 days postinoculation, the colonizing bacteria were evaluated by microscopy analysis, and root samples were cut, soaked in sterilized H_2_O, placed on glass slides with PBS and covered with glass coverslips. Confocal images were obtained using a Nikon C2 + CLSM (Nikon, Tokyo, Japan) equipped with a CFI Plan Apo Lambda 20× objective and two helium–neon lasers for the excitation of the mCherry fluorophore at wavelengths of 540 nm and 650 nm and an argon laser for the excitation of autofluorescence at 488 nm. The cell morphology and localization of the *A. brasilense* strains (sample and control) were evaluated in the roots to assess the presence of the bacteria and biofilms.

### *cdgD* gene promoter expression in wheat plants determined by confocal microscopy

Nana wheat seeds were sterilized as described in the previous section and germinated on sterile 1.0% H_2_O-agar for 72 h in the dark. The seedlings were then transferred to FluoroDish glass-bottom Petri dishes containing Hoagland hydroponic solution and placed in an environmental chamber (24 °C, 14 h light and 16 °C, 10 h dark photoperiod). The *A. brasilense* T7mCh and *A. brasilense* FPDm1 strains were grown overnight in LB* liquid medium, and the cultures were subsequently diluted in phosphate buffer and used to inoculate 3-day-old seedlings at 1 × 10^7^ CFU/mL. The samples were visualized by confocal microscopy for 24 or 48 h to evaluate *cdgD* gene promoter expression in association with wheat plants. Confocal images were acquired using a Nikon C2 + CLSM (Nikon, Tokyo, Japan) equipped with a CFI Plan Apo Lambda 20× objective and two helium–neon lasers for the excitation of the mCherry fluorophore at wavelengths of 540 nm and 650 nm and an argon laser for the excitation of autofluorescence at 488 nm. The Nikon NIS Elements program provided by Nikon was used to acquire, analyse, and edit the images.

### Plant colonization experiments under soil conditions

Wheat seed disinfection and the inoculation of seedlings 3 days after germination were performed as described above. The seedlings were placed in an environmental chamber maintained under a day–night cycle as described above for 3 days. Next, the seedlings were inoculated with 1 × 10^6^–1 × 10^7^ CFU/mL of the *A. brasilense* Sp245, *A. brasilense* 12-A, *A. brasilense* C-56A or *A. brasilense* C-40A strain, grown in a 50 mL flask with sterile soil for 7 days postinoculation and handled as described above. Subsequently, the plants were carefully washed to remove all adhered soil with sterile water for 40 s. The colonizing bacteria were quantified by colony counting in CR medium supplemented with the appropriate antibiotic (WT, Ap, 150 µg/mL; *A. brasilense* 12-A, Km, 50 µg/mL; C-40A or C-56A, Tc, 20 µg/mL).

### Statistical analyses

For all experiments, the overall data were analysed by Student’s t-test, to determine whether the mutant significantly affected all phenotypes tested and the expression of *cdgD* gene under the analysed growth conditions as well as the colonization tests. The difference were considered to be significant when the *p* value were < 0.05. Statistical analyses were performed using Sigma plot 11.0 software (Systat software, Sn Jose, CA).

## Results and discussion

### Homology modeling and in silico analysis

While biofilm formation is often described as a natural mode of microbial growth and the second messenger c-di-GMP is known to be a central molecule regulating the microbial transition from a planktonic (motile) to sessile (biofilm) lifestyle^4,15,38^, it is clear that we still lack knowledge regarding the genes encoding the proteins responsible for cyclic-di-GMP biosynthesis and degradation and the roles that they play in host plant colonization by *A. brasilense*.

**Figure 1:**
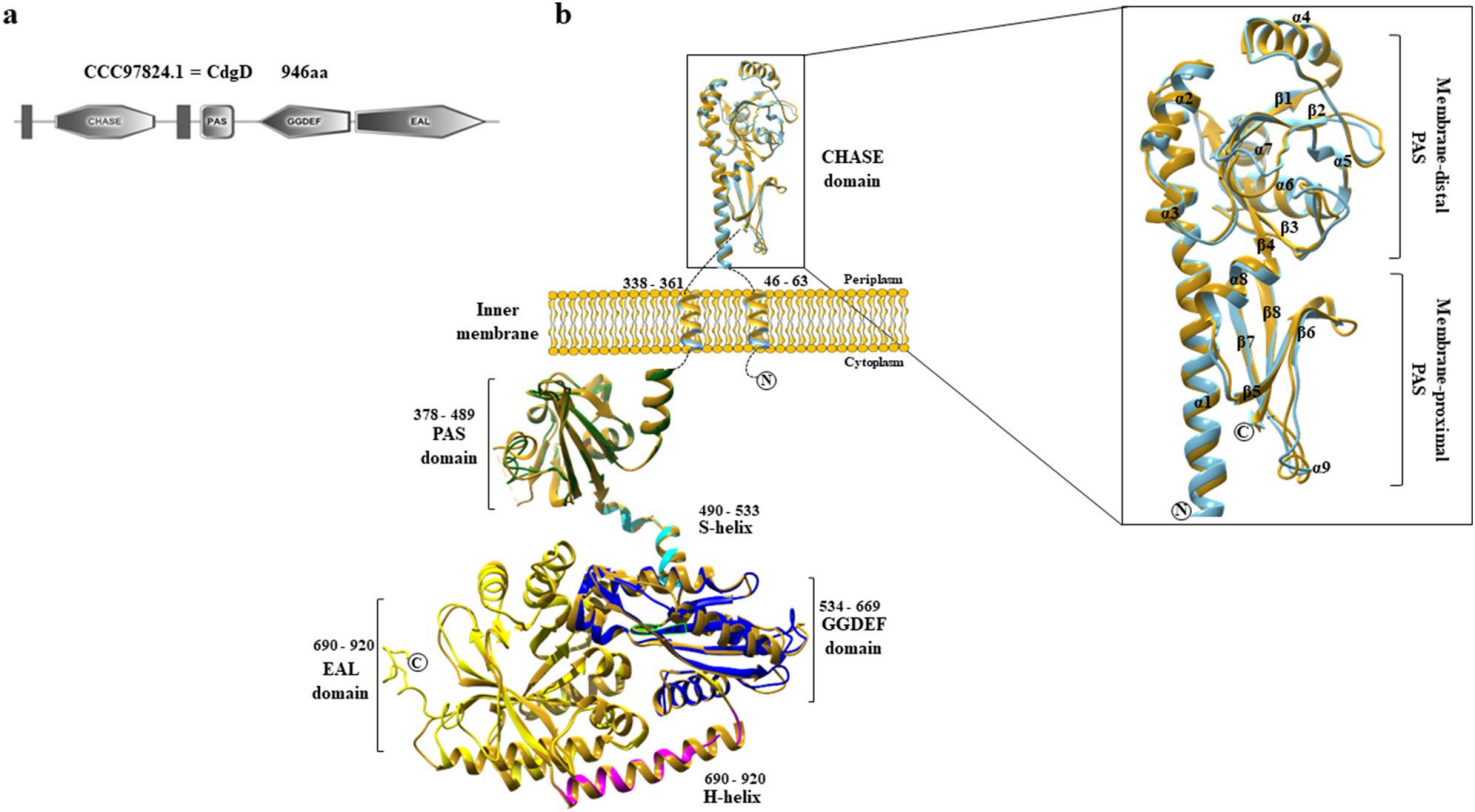
Structural architecture, domain organization, and homology modeling of the CdgD protein of *A. brasilense* Sp245. (**a**) CdgD domains determined by the SMART domain prediction server, with a sequence length of 946 amino acid residues. (**b**) A representation of the CdgD protein with its CHASE sensory domain, two transmembrane domains (TMD), PAS domain, GGDEF domain, EAL domain, the S-helix and H-helix connecting segments crucial for protein dynamics, and key structural features. Overlay of the structures of the periplasmic sensing domains and transmembrane domains of CdgD showing the CHASE domain of PcrK (histidine kinase from *X. campestris* pv *campestris,* PDB code 6K62) in gold and the CHASE domain of CdgD in light blue. cRbdA from *P. aeruginosa* (PDB code 5XGB), shown in gold, was compared with the cytoplasmic region of CdgD. The PAS domain is green, the GGDEF domain is blue, the EAL domain is yellow, and the S-helix and H-helix are cyan and magenta, respectively. The loops that are not included in the structural model are represented schematically by dashed lines. The N and C termini are labeled. Magnification of the CHASE domain showing the membrane-distal PAS subdomains and membrane-proximal PAS subdomains, in which the α-helices and ß-strands are indicated. CdgD and PcrK are light blue and gold, respectively.

An initial analysis was conducted to confirm the domains constituting the translational product encoded by the *cdgD* gene (GenBank accession number AZOBR_100210) and further used for structural modeling. Alignments were generated and analysed using the Clustal Omega server (www.ebi.ac.uk/Tools/msa/clustalo/) against equivalent domains known to be catalytically active for which a structure has been deposited in the PDB; this approach employed to compare the motifs of the translational product of the *cdgD* gene to the WspR of *Pseudomonas aeruginosa* or the PleD of *Caulobacter crescentus* diguanylate cyclase as a reference for the amino acid motifs of the GGDEF protein domain^39,40^ and to RocR from *P. aeruginosa* as a reference for the EAL-containing domain^41^ to identify the GGDEF and EAL domains in the CdgD hybrid protein. The identified GGDEF domain consists of highly conserved sequence motifs that include the D/E catalytic site and other residues involved in the substrate binding and coordination of one of the two divalent cations^41^. Moreover, we identified a motif acting as a regulator of DGC function, referred to as the I site, which was determined to contain an RXXD motif immediately preceding the GGDEF catalytic motif^42^ (Supplementary Fig. S2a,b). The EAL domain also exhibits all characteristics of the amino acid motif required for the binding of divalent cations and the substrate. In addition, a flexible loop (loop 6) that mediates dimerization and controls substrate and cation binding, which are required for catalytic activity, was also found^42,43^, (Fig. 1 and Supplementary Fig. S2a,b). Notable characteristics were also observed in the cytoplasmic region of CdgD, including an α-helical lever (H-helix) that joins the GGDEF and EAL regions as well as a signaling α-helix (S-helix) located immediately adjacent to the GGDEF domain (Fig. 1), which has been shown to be important in the folding and regulation of PDE function of RbdA from *P. aeruginosa*^32^. Therefore, we might categorize both domains as functional based on the presence of conserved amino acid motifs in the active sites of the two domains^11,12^. Moreover, the CdgD protein contains three other domains at the N-terminus, including two transmembrane (TMD) domains, a CHASE domain, and a PAS domain, which is a sensor domain that modulates activities in response to external stimuli such as light or small ligands, including O_2_, NO, and CO^44^. This PAS domain was identified using the structure of the cytoplasmic region (cRbdA) of RbdA from *P. aeruginosa* as a model that exhibits a highly conserved structural architecture in the cytoplasmic region of the CdgD protein (residues 241–557; PDB Code: 5XGB, resolution 2.28 Å, TM-score 0.918, RMSD 0.79 Å)^32^, (Supplementary Table S3, Figs. S3, S4, S5 and S6).

It has been reported that these GGDEF and EAL domain-containing proteins often harbor a diverse array of additional domains that are important for the biochemical function of the specific protein, such as a CHASE domain, which is a periplasmic domain that is defined as a sensory domain and predicted to play a role in small molecule recognition in a wide range of proteins and various bacterial chemotaxis receptors^45,46^ and may be a ligand-binding domain (LBD)^47^, (Fig. 1). This CHASE domain was located in the N-terminus of the protein between two TMDs (including 264 amino acid residues); according to the presence of these predicted transmembrane segments, the protein may be associated with the cytoplasmic membrane. Structural analysis of the CHASE domain was performed using the same domain found in *Xanthomonas campestris* pv *campestris* histidine kinase PcrK (residues 39–302; PDB Code: 6K62, resolution 2.55 Å, TM-Score 0.955, RMSD 0.82 Å)^30^ as a reference model (Fig. 1 and Supplementary Table S3, Figs. S3a,b and S4a,b). The structure showed the highest-confidence resolution scores (TM 0.928 and RMSD 0.74 Å) and fit well with the CHASE domain, and it was recognized as a structural homologue of the CdgD protein, a notably similar structure showing the predicted formation of a homodimer between the two TMDs, with the predicted function of a binding domain involved in periplasmic signaling (Fig. 1). Such a situation has also been described for the *Arabidopsis thaliana* histidine kinase (AHK4) 4–10 protein (PDB Q9C5U0)^31^, which has been experimentally shown to be able to bind with cytokinin and both models to provide structural patterns that match the CHASE domain of the CdgD protein well. It was also predicted that the CHASE domain is composed of two PAS domains (membrane-distal and membrane-proximal PAS domains) displaying the same numbers of ß-strands and α helices, even though CdgD sharing only 25% and 19% sequence identity with PcrK or AHK4, respectively (Fig. 1). These results suggest that the CdgD sensor domain adopts a conserved CHASE topology overall, which is consistent with previous findings^30,31^. The occurrence of CHASE and PAS domains has been observed in other hybrid proteins, in which the module mediating the signaling pathway has an effect on the regulation of the dual function of DGC-PDE proteins^48,49^.

Therefore, the high conservation of all these motifs in GGDEF and EAL domains, as determined through the sequence alignment and analysis of the three-dimensional (3D) structure predicted for the translational product of the *cdgD* gene suggest that it is likely bifunctional and may be classified as a class I CdgD hybrid protein^11^. To verify this hypothesis, an insertional *cdgD::Km*^*R*^ mutant was constructed (designated as *A. brasilense* 12-A) to test the functions of DGC and PDE to gain further systematic insight into the effect of the mutation on certain phenotypic characteristics (Supplementary material Table S1).

### Phenotypic studies of the *cdgD* gene

#### Biofilm formation and extracellular polymeric substance (EPSs) production

**Figure 2:**
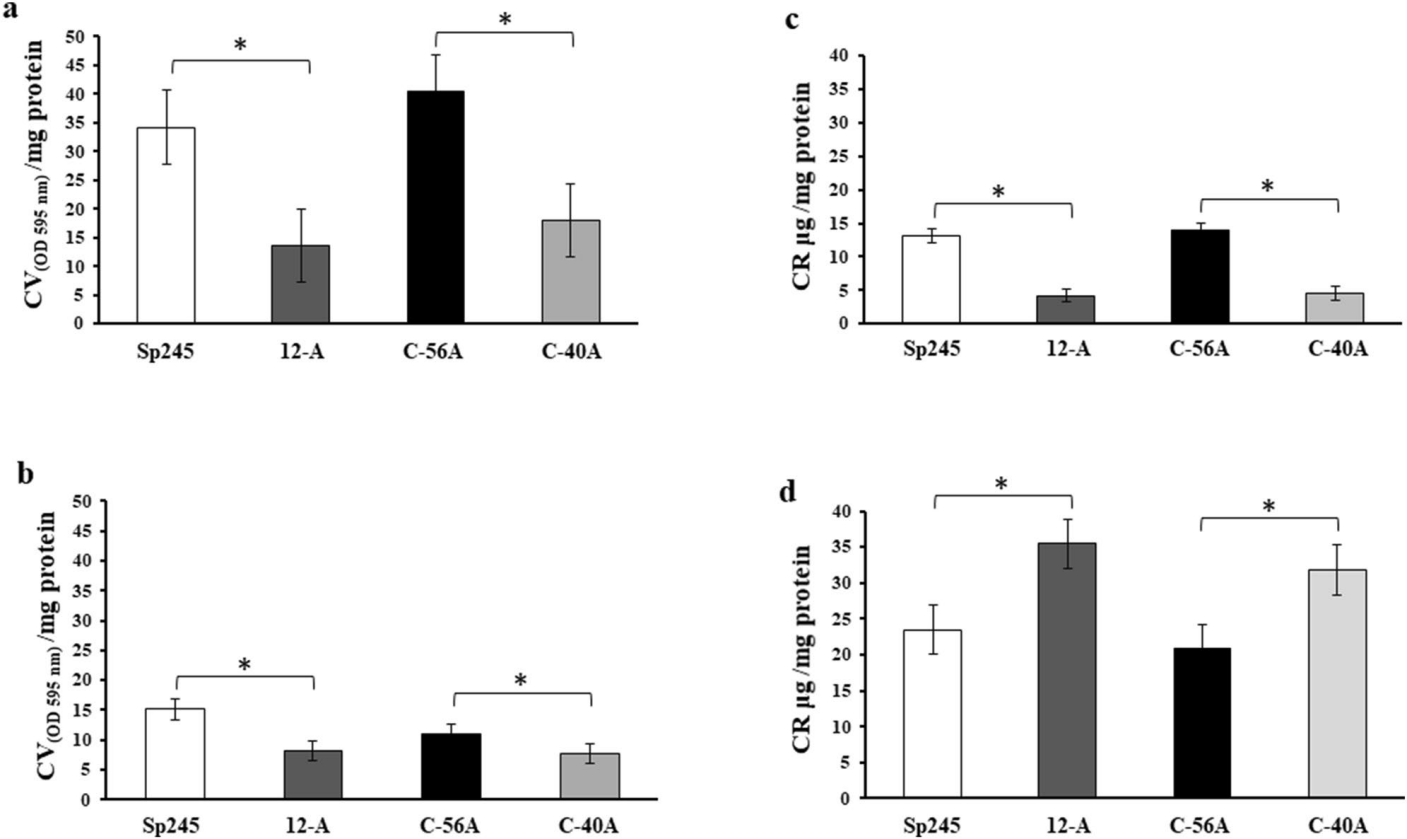
Effects of the mutation of the *cdgD* gene on biofilm and EPS production determined in the *A. brasilense* WT and derivative strains. (**a**) Determination of biofilm formation in NFB* medium supplemented with KNO_3_ via the quantification of CV/mg protein values. (**b**) Determination of biofilm formation in NFB* supplemented with NH_4_Cl medium via the quantification of CV/mg protein values. (**c**,**d**) Determination of EPS production in the media described above via the quantification of CR/mg protein values in a staining assay. Wild type (Sp245), isogenic mutant (12-A), complemented mutant (C-56A), and empty vector mutant (C-40A). Error bars represent the standard deviations of three biological replicates, and statistically significant differences are indicated at *P < 0.05 according to Student’s t-test by SigmaPlot (Systat Software, San Jose, CA).

To elucidate the molecular basis of the role of the *cdgD* gene of the *A. brasilense* Sp245 strain in biofilm formation, motility and the colonization of wheat roots, we constructed the *A. brasilense* 12-A mutant. Sequence analysis of this mutant showed that the insertion of the Km resistance cassette interrupted the ORF of the translational product. Additionally, we constructed a corresponding complemented mutant strain harboring the wild-type *cdgD* gene to compare the effect of the mutation on biofilm formation and motility against the WT strain. The different strains and plasmids that were constructed and used in this study are shown in Supplementary Table S1. To characterize the differences between *A. brasilense* Sp245 and the derived strains, the strains were grown in NFB* medium supplemented with two different nitrogen sources and cultured under static conditions. Under such conditions, we observed that biofilm formation by the WT was greater in NFB* + KNO_3_ than NFB* + NH_4_Cl medium (Fig. 2a,b). However, when the bacteria were grown in each type of nitrogen NFB* medium, a marked decrease in the production of biofilm was observed in the mutant compared with the WT, which was recovered in the complemented strain.

Extracellular polymeric substances have been reported^4,6,50,51^ to play essential role in the formation of the biofilm matrix. To quantify extracellular polymeric substances such as exopolysaccharides, extracellular DNA (eDNA) and amyloid compounds, we tested their capacity to bind the colorant Congo Red (CR)^8,51–54^, and the production of these components was assessed in the strains growing in NFB* medium supplemented with either KNO_3_ or NH_4_Cl. As anticipated, the mutant growing in NFB* + KNO_3_ medium showed a decrease in EPSs production (3.2-fold reduction) compared with the WT, whereas EPSs production similar to that in the WT strain was observed in the complemented strain (Fig. 2c). This data showed that the decreases in biofilm production and extracellular polymeric substance production observed in the mutant strain were corelated in NFB* + KNO_3_ medium (Fig. 2a,c). The mutation of the predicted CdgD, which contained a highly conserved GGDEF motif, decreased biofilm formation and affected the production of EPSs matrix components, as determined by quantification based on staining with CV^53^ and CR colorant binding to components of the matrix structure of biofilms^51,54^, this results are consistent with this protein being a DGC. However, an unexpected result was obtained when the strains were grown in NFB* + NH_4_Cl medium. In effect, the mutant showed an increase in EPS production of 1.6-fold compared with the WT strain (Fig. 2d). In contrast to the data obtained on biofilm formation, in the case of extracellular polymeric substance production, a significant increase was notably observed in the mutant in medium supplemented with NH_4_Cl. Furthermore, when the EPSs quantification data for the WT strain were compared to analyse growth in NFB* + KNO_3_ and NFB* + NH_4_Cl (13.13 µg/mg protein compared with 23.4 µg/mg protein, respectively), 1.7-fold higher production was observed in the medium supplemented with ammonium (Fig. 2c,d). Similar results were obtained for cells of the 12-A mutant growing under the same conditions (4.1 µg/mg protein versus 37.3 µg/mg protein), corresponding to 9.1-fold higher production. These data indicated that the nitrogen source causes changes in cell surface properties that contribute to CR binding (Fig. 2c,d). Therefore, the data obtained in each type of nitrogen-supplemented NFB* medium suggest a differential contribution of the *cdgD* gene to cell surface properties, as demonstrated by changes in binding to CR. These results are notable because amongst the EPS, the production of extracellular polysaccharides in flocs plays an important role in the infection bacterial ability to colonize the surface of plant roots^2,15,54–57^. Furthermore, the nitrogen source had an effect on biofilm formation, which was more significant in presence of KNO_3_ compared to NH_4_Cl (Fig. 2). These data are in keeping with findings obtained in both *A. brasilense*^58,59^ and other bacterial species such as *Pseudomonas stutzeri* A150^52^ showing that the nitrogen source affects the formation of biofilms and the production of EPSs, particularly the exopolysaccharide components that allow nitrogen fixation by the bacterium^52,59^. Moreover, in *Cellulomonas* spp., nitrogen source limitation induces biofilm formation, which is coupled to the biosynthesis of a curdlan-type biofilm matrix. However, when a nitrogen source is available, the biofilm curdlan matrix is solubilized and used as a carbon and energy source^60^. In *A. brasilense*, different classes of exopolysaccharides are produced under high carbon source and low nitrogen source availability, which were the conditions applied in this study^56,58^. Furthermore, in terms of functional redundancy, several genes presumably encoding DGCs, PDEs or DGC-PDEs have been described in *A. brasilense*^12,21^, this gene products could provide compensatory effects that could be responsible for the production of different exopolymeric substance contributing to the changes in cell surface properties observed in cultures grown in medium supplemented with NH_4_Cl as a nitrogen source. It is also possible components of the biofilm matrix in the single-gene mutant are directly or indirectly upregulated in the *A. brasilense* 12-A mutant, as described in other beneficial plant bacteria^7,13,14^.

#### Motility assay

**Figure 3:**
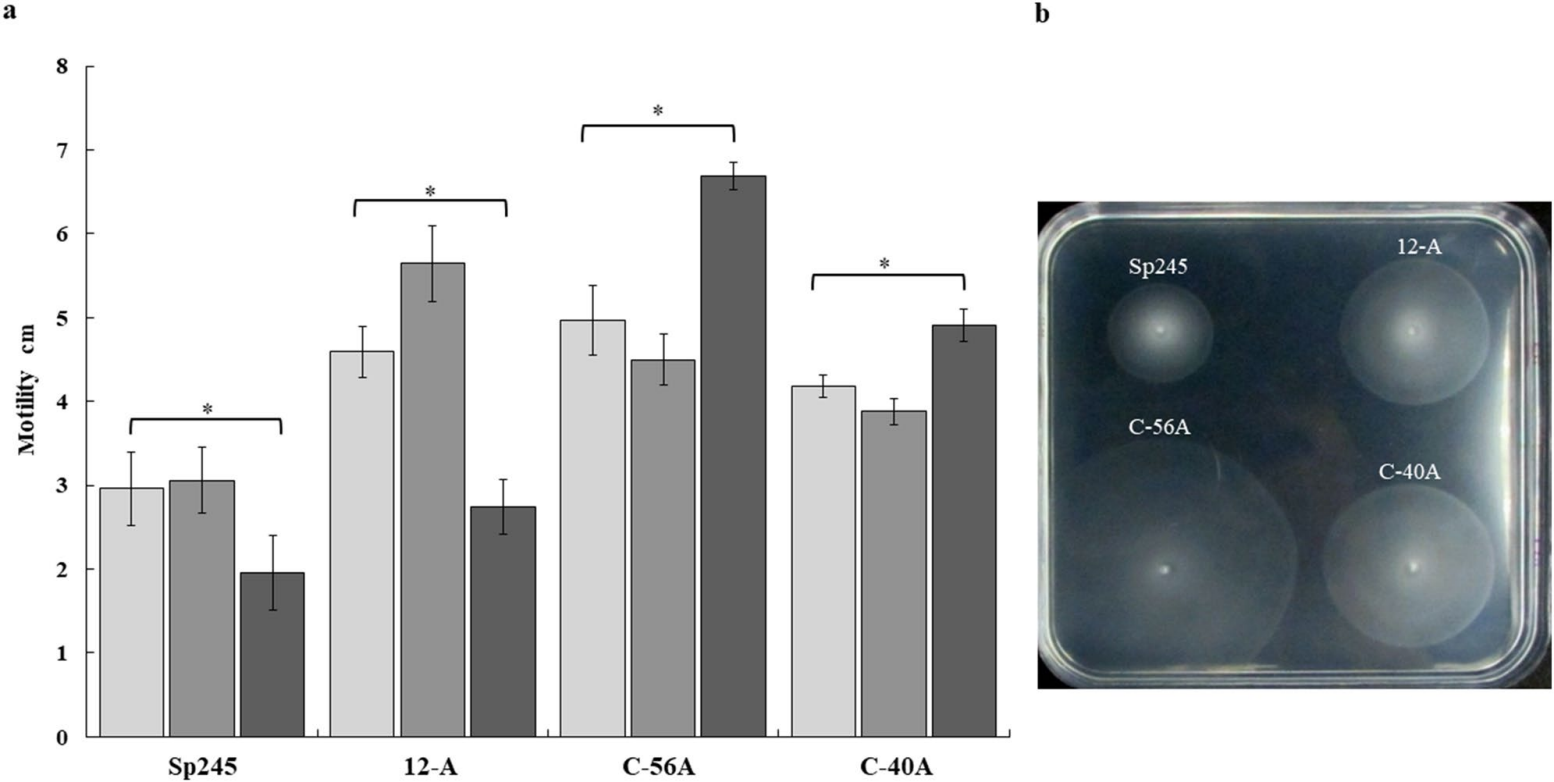
Effects of the mutation and overexpression of the *cdgD* gene on motility determined in *A. brasilense* WT and derivative strains. (**a**) The diameter of the swimming rings in the mutant and the complemented mutant relative to the wild-type strain was measured in minimal medium with the tested carbon sources at 10 mM after 48 h of incubation at 30 °C ( malate,  fumarate, or  proline). Error bars represent the standard deviations of three biological replicates, and the asterisks indicate values that are significantly different from those in the wild type (*P* < 0.05) according to Student’s T test by SigmaPlot (Systat Software, San Jose, CA). (**b**) Photograph of a typical agar plate containing minimal medium with 10 mM proline as the carbon source showing the swimming rings of each strain. Wild type (Sp245), isogenic mutant (12-A), complemented mutant (C-56A), and empty vector mutant (C-40A).

Next, we tested the ability of the 12-A mutant and the complemented C-56A mutant to swim in semisolid agar minimal medium supplemented with malate, fumarate or proline as a carbon source. All strains had been previously tested in NFB* + KNO_3_ medium and showed similar growth (Supplementary material Fig. S7). All rings sizes formed by the 12-A mutant or the C56-A are larger than those formed by the wild type strain in all carbon sources tested, however when added to the K-minimal medium, the effect of proline was more pronounced (3.4 times greater than in the WT strain) (Fig. 3a,b).The constructed complemented mutant was generated in the intermediate-copy-number vector pVK100, under which condition the *cdgD* gene was overexpressed and cells grown on soft agar plates exhibited increased swimming motility. Furthermore, cells grown in broth medium exhibited distinct swimming patterns, and the cells of WT strain reduced their motility and began to form small flocs, whereas cells from 12-A mutant or complemented mutant C56-A (Supplemental Movies S1, Movie S2, Movie S3 and Movie S4) continued to move. It has been reported that aerotaxis is a major behavioral response in *A. brasilense*, and the bacterium depends on sensing oxygen gradients. It has also been shown that flocs are formed when oxygen is increased^3,59,61^. Therefore, we suggest that WT cells sensed oxygen more efficiently than the 12-A and C56-A cells did and that they continuously moved mostly without changing the direction of swimming; however, that behavior warrants further research. In addition, it has also been reported that motile cells of *A. brasilense* undergo chemotaxis towards several nutrients found in root exudates, which has been shown to be an essential prerequisite for efficient root colonization^61^. CHASE domains have been observed to sense environmental signals, such as chemoattractants, including organic acids, amino acids and other compounds that are found in root exudates^62,63^. The ability of proline to increase *A. brasilense* motility might be due to the presence of the CHASE domain, which is also able to bind amino acids and other small molecules^47^. Alternatively, other DGCs/PDEs may produce the phenotype in the absence of *cdgD* and perhaps even amplify the effect of CdgD on C-56A. Nevertheless, future studies will clearly establish the nature of the signal recognized by the predicted ligand-binding domain (LBD) of the CHASE domain and elucidate the ligand and signal transduction mechanism.

#### Determination of cellular cyclic-di-GMP levels using a riboswitch biosensor

**Figure 4:**
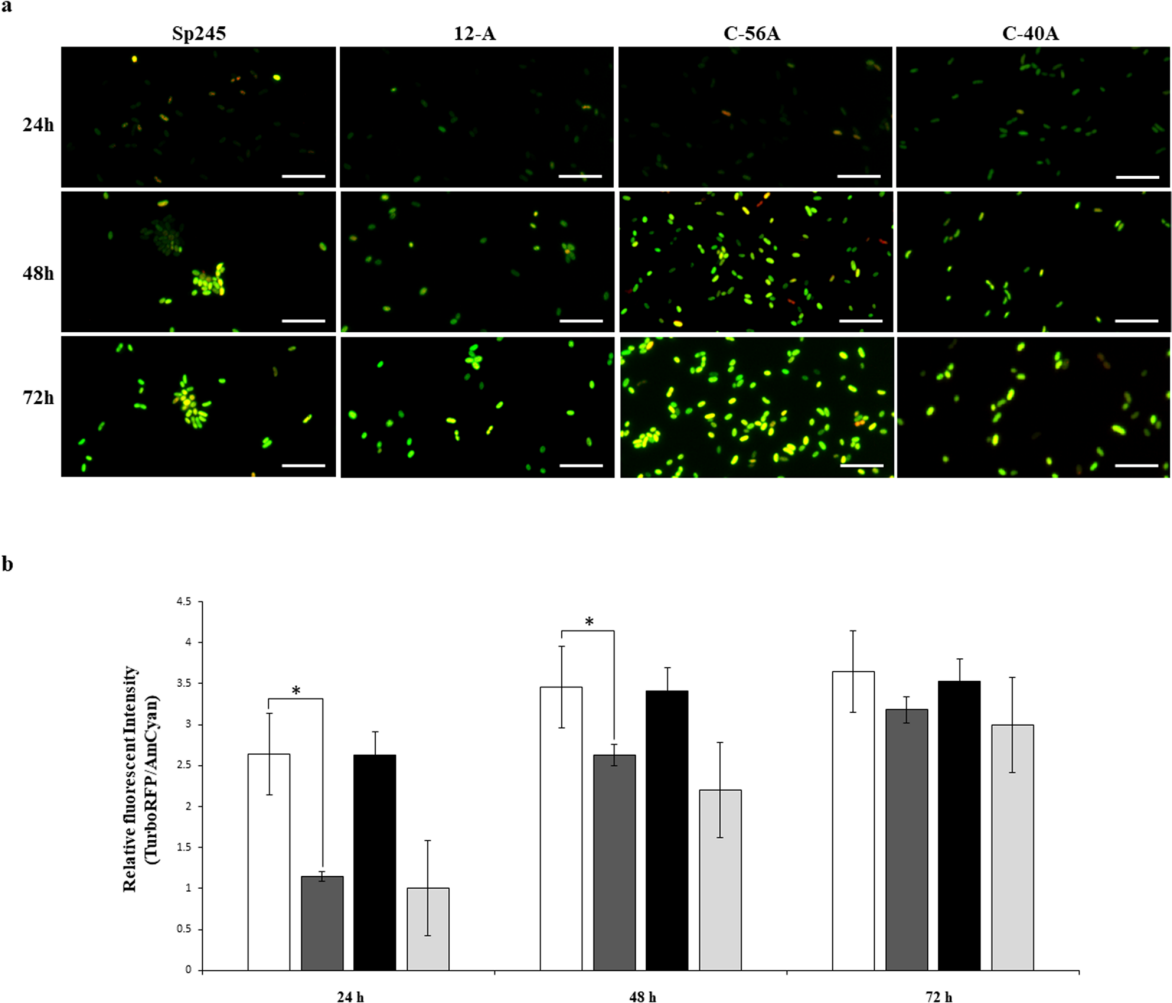
Determination of cyclic-di-GMP levels using a riboswitch biosensor. (**a**) The *A. brasilense* Sp245  , *A. brasilense* 12-A  , and *A. brasilense* C-56A  , and C-40A  strains containing the c-di-GMP biosensor (pFY4535) were grown in NFB* + KNO_3_ broth containing 30 μg/mL Gm with incubation for 24, 48, or 72 h at 30 °C. Then, *Azospirillum* cells were attached to the surface of a coverslip and sealed with a 1% agar plug. Cell images were collected at these time points after inoculation using a Nikon TE2000U microscope equipped with a 100× objective (oil immersion objective). Merge images represented the overlay of the fluorescence images AmCyan green, TurboRFP red and both yellow. (**b**) RFI represents the ratio between the TurboRFP and AmCyan fluorescence intensities and is directly proportional to c-di-GMP levels, as analysed using ImageJ software^35^. The RFI values represent the standard deviations of three biological replicates, and significant differences are indicated at *P < 0.05 according to Student’s t-test by SigmaPlot (Systat Software, San Jose, CA). The bar corresponds to 10 µm.

Taken together, the results suggest that CdgD is capable of c-di-GMP synthesis, thereby validating its contribution, and indicate that this function is important for biofilm formation and might modulate extracellular polymeric substance production. We estimated the intracellular levels of c-di-GMP in the wild-type (Sp245), 12-A mutant, C-40A control carrying out the vector empty and complemented mutant (C-56A) strains using a fluorescent c-di-GMP reporter. This analysis was performed by quantifying the riboswitch fluorescence intensity, which was directly proportional to the c-di-GMP levels^34^. As expected, a low c-di-GMP level was determined in the 12-A mutant compared with the wild-type strain; an approximately 61% or 24% reduction in c-di-GMP levels was observed in the 12-A mutant strain compared with WT cultures at 24 h or 48 h, respectively, which was fully recovered in the complemented mutant. However, there was no significant difference in intracellular c-di-GMP levels compared with the WT strain in the cells after 72 h of growth, potentially due to compensatory effects of other DGCs occurring in the genome of the *A. brasilense* WT strain. In accordance with the data regarding biofilm and EPSs component production from the CR assay, the mutation reduced the levels of c-di-GMP in cells growing in NFB* + KNO_3_ medium. These data indicate that overexpression of CdgD allows a temporary intracellular second messenger levels in *A. brasilense* (Fig. 4). Although bioinformatic analysis showed that all amino acid motifs were conserved in the EAL domain, the phenotyping studies were unable to show that CdgD functioned as a PDE protein.

#### Expression of the *cdgD* gene under abiotic and biotic conditions

**Figure 5:**
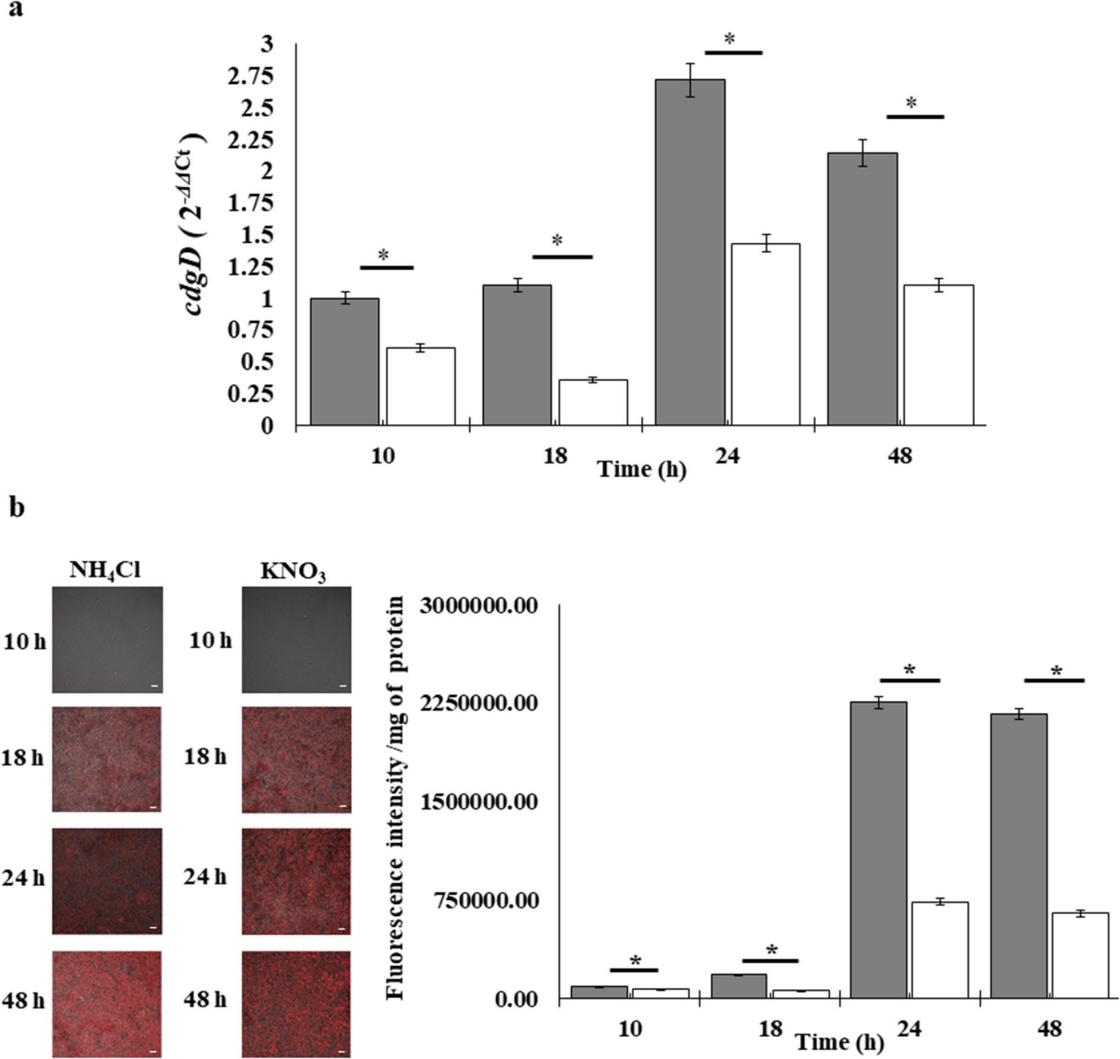
Expression analysis of the *cdgD* gene from planktonic and static cultures of the *A. brasilense* Sp245 strain. (**a**) Determination of *cdgD* gene transcript levels by RT-qPCR. The transcript level data were normalized to those of the reference gene *glyA* and determined relative to the control sample using Applied Biosystems StepOne software. (**b**) Fluorescence micrographs of the *A. brasilense* FPDm1 strain under different culture conditions. Left, bacteria grown in NFB* + KNO_3_ media; right, bacteria grown in NFB* + NH_4_Cl media. The fluorescence intensity is normalized to total protein values from the *A. brasilense* FPDm1 strain, as analysed using ImageJ^35^. The presented data are the results of three independent experiments with two biological replicates, and the asterisks indicate significant differences from the wild type (*P* < 0.05) according to Student’s T test by SigmaPlot (Systat Software, San Jose, CA). The bar corresponds to 10 µm.  NFB* + KNO_3_,  NFB* + NH_4_Cl.

Next, we monitored *cdgD* expression by RT-qPCR in exponential- and stationary-phase cultures grown in NFB* supplemented with KNO_3_ or NH_4_Cl under shaking (10 and 18 h) and static conditions (24 and 48 h) and by using the tagged chromosomal *A. brasilense* FPDm1 strain and *A. brasilense* T7mCh, carrying the *mCherry* gene devoid of the promoter as a negative control. Both strains showed similar growth to the wild-type strain, as determined by the 10^9^ CFU/mL value obtained in the exponential phase of growth (18 h), (Fig. 5). In both media, the expression of *cdgD* was initiated at 10 h and continued until 48 h, in cultures growing in planktonic and static conditions. These observations indicate that the expression of the gene is initiated at the beginning of bacterial growth and continues until the later stages of growth. The amount of fluorescence produced was higher in cells growing in NFB* + KNO_3_ medium comparative to NFB* + NH_4_Cl, (Fig. 5b), indicating that the addition of KNO_3_ to the medium increased transcript levels relative to those obtained in presence of NH_4_Cl, where expression was further increased when the structure of the biofilm was structurally mature (48 h) (Fig. 5a). Such data indicate a role of the nitrogen source in determining gene expression, which may be direct or indirect. In addition, the data were correlated well with both biofilm production (Fig. 2a) and the production of extracellular polymeric substances (Fig. 2c).This suggests a function for CdgD as a DGC under static culture conditions, which is also supported by elevated cellular c-di-GMP levels (Fig. 4). Considering the data obtained in this study, we suggest that the regulation of the *cdgD* gene by nitrogen source might occur at the transcriptional level with CdgD protein activity as either a DGC or PDE or regulated at the post-translational level, as observed in other bacteria^10,13,14^. However, how signaling involving the CHASE domain modulates the DGC-PDE functions of the CdgD protein remains to be studied.

**Figure 6:**
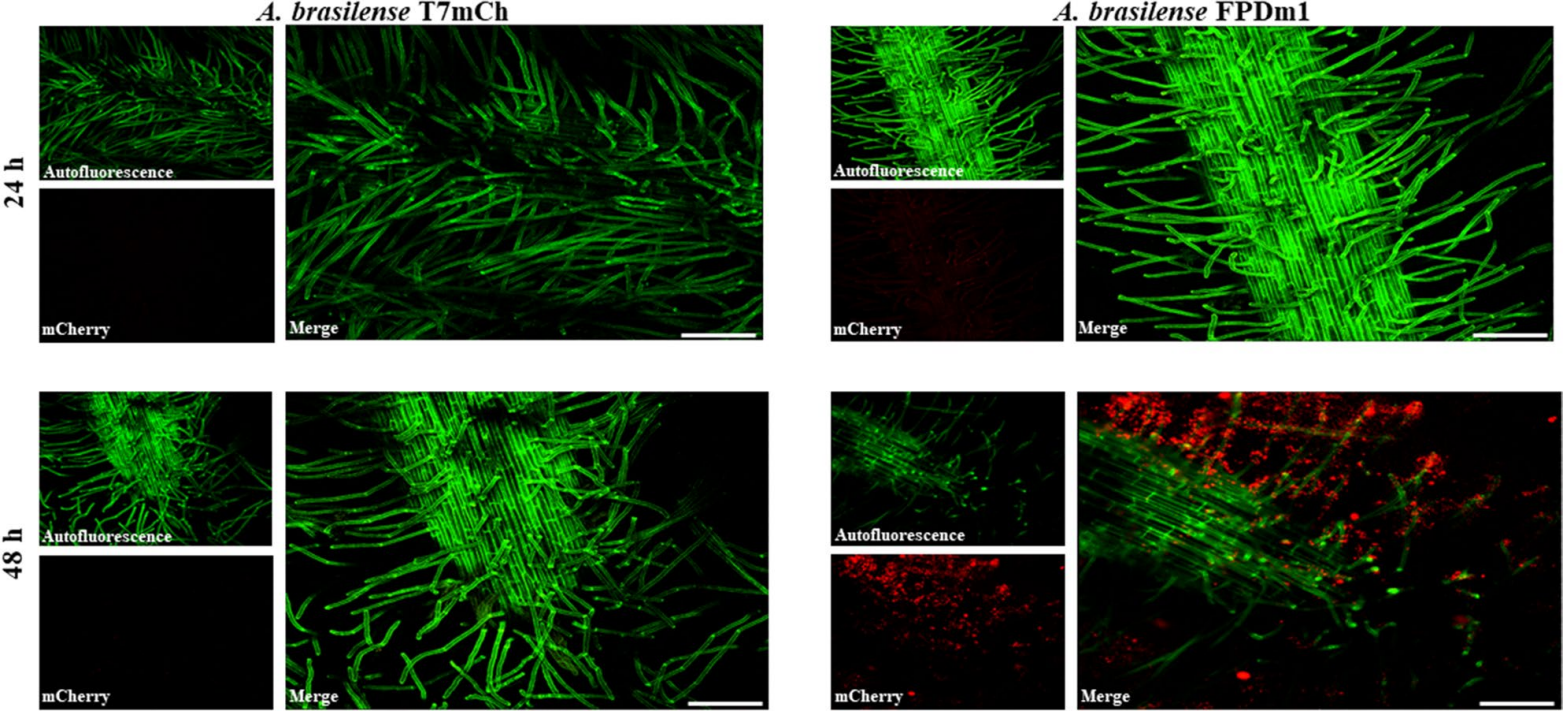
Expression of the *pcdgD-mCherry* transcriptional fusion from *A. brasilense* cells grown under hydroponic conditions and in wheat roots. For the visualization of *mCherry*-expressing *A. brasilense,* cells on wheat roots were observed at 24 h and 48 h postinoculation. Next, the freshly harvested seedling roots were visualized in a FluoroDish. Left, wheat seedling roots inoculated with the *A. brasilense* T7mCh control strain. Right, wheat seedling roots inoculated with the *A. brasilense* FPDm1 strain. The cells exhibiting mCherry fluorescence were visualized using a CLSM with an excitation wavelength of 561 nm, with fluorescence emission captured between 585 and 615 nm. Thereafter, the images were edited using the standard NIS Elements in Nikon software. The bar corresponds to 50 µm.

Next, we attempted to monitor *cdgD* gene promoter expression during the colonization of wheat roots under hydroponic medium conditions by using the tagged chromosomal *A. brasilense* FPDm1 strain and *A. brasilense* T7mCh, carrying the *mCherry* gene devoid of the promoter as a negative control. Both strains showed similar growth to the wild-type strain, as determined by the 10^9^ CFU/mL value obtained in the exponential phase of growth (18 h). To visualize the localization of CdgD in vivo, we expressed the m*Cherry* gene fused upstream of the promoter of the *cdgD* gene. In effect, this hypothesis was verified because *A. brasilense* FPDm1 cells that were recovered following 48 h of exposure to the root environment of wheat seedlings showed significant fluorescence in cells maintained on root hairs, and rhizoplane-forming microcolonies were observed, which represent a preliminary step in biofilm formation; zones exhibiting the formation of a mature biofilm were also observed, indicating the expression of the *cdgD* gene, which was clearly lacking in *A. brasilense* T7mCh (Fig. 6). These findings suggest that the activity of the encoded protein is relevant to the early stages (48 h) of colonization in our model rhizosphere assay and that the bacterium is able to enter the roots and thereby colonize the apoplast tissue of wheat roots. In addition, our data suggest that biofilm formation is stimulated as a response to wheat rhizosphere colonization (Fig. 6). This results is in keeping with data obtained in the plant-growth-promoting strain *Pseudomonas fluorescens*, in which genes involved in the metabolism of c-di-GMP are expressed during the colonization of the wheat rhizosphere^14,38^, as well as data from *Pseudomonas putida* KT2440, in which the promoter of the *rup4959* gene, encoding a dual GGDEF-EAL protein, was found to be expressed in the maize rhizosphere and upregulated by corn plant root exudates^13^.

**Figure 7:**
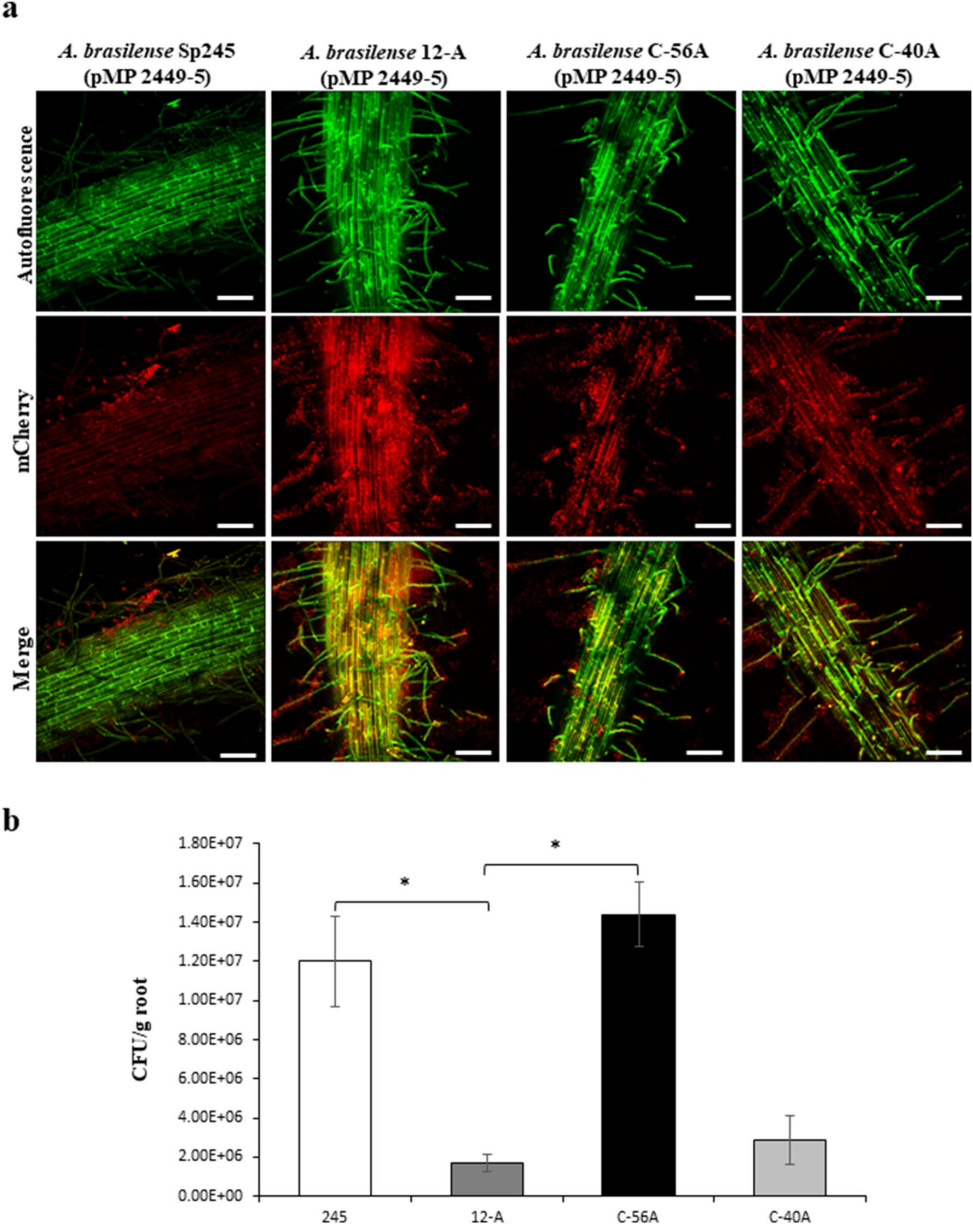
Rhizosphere colonization of *A. brasilense* Sp245, the 12-A mutant, the C-40A control mutant and the C-56A complemented mutant and visualization of tagged colonizing bacteria by CLSM. (**a**) The plants were grown in glass tubes with Hoagland hydroponic solution and examined at 7 days postinoculation with 5 × 10^6^–10^7^ CFU/mL of *A. brasilense* Sp245 (pMP2449-5), *A. brasilense* 12-A (pMP2449-5), *A.brasilense* C-40A (pMP2449-5) and *A. brasilense* C-56A (pMP2449-5) (the WT, mutant, and complemented mutant strains, respectively). Subsequently, the cells showing mCherry fluorescence were visualized using CLSM with an excitation wavelength of 561 nm, with fluorescence emission captured between 585 and 615 nm. Thereafter, the images were edited using the standard NIS elements in Nikon software. The bar corresponds to 10 µm. (**b**) The bacterial colonies recovered from the rhizosphere 1 week after inoculation under sterile soil conditions were tested for antibiotic resistance to distinguish the control and mutant strains and were counted. CFU/mL per gram of plant root values are presented on a logarithmic scale. Each experiment was repeated three times with five plants per experiment. Asterisks represent the statistical significance of the data (*P* < 0.05) according to Student’s t-test by SigmaPlot (Systat Software, San Jose, CA).

We further tested the role of the *cdgD* gene in the colonization of wheat roots, and we estimated endorhizosphere colonization by the *A. brasilense* 12-A mutant and compared it with colonization by the WT and complemented *A. brasilense* C-56A mutant strains. Each strain was labeled with the autofluorescent mCherry protein, which was carried by a stable plasmid maintained in *A. brasilense* cells^37^. The plants were grown in glass tubes with Hoagland hydroponic solution and observed by confocal microscopy at 7 days postinoculation. Microscopy observations of these reporter strains indicated that all strains were able to colonize the rhizoplane and root hairs of wheat plants and form biofilms (Fig. 7a). However, differential colonization was visualized in the *A. brasilense* 12-A mutant and its C-40A control strain harboring the empty vector, which exhibited significant rhizosphere colonization. Next, the colonization of the *A. brasilense* 12-A mutant was quantified under two conditions: under hydroponic growth and in sterile soil and compared to the fitness of the WT and the complemented mutant. The colonization of each strain was determined by counting the colonies obtained at 5 days postinoculation. Nonsignificant differences in fitness and the impairment of colonization were determined in the tested strains under most controlled hydroponic conditions (data not shown). However, the *A. brasilense* 12-A mutant and its control strain harboring the empty vector showed a statistically low ability to colonize wheat roots compared with the WT and complemented mutant C-56A strains tested in sterile soil (Fig. 7b), implying that the *cdgD* gene is involved in the colonization of wheat roots.

However, in our study, the cells of the tagged mutant were observed more frequently than those of the WT and complemented mutant strains in the rhizoplane and rhizosphere (Fig. 7a). This result was surprising because *A. brasilense* Sp245 is an endophytic strain that has been found in the apoplast of plants^63,64^. A different colonization profile was observed in wheat roots associated with the 12-A mutant strain versus the Sp245 or C-56A strain. This effect could be explained by considering that cellulolytic activity might be crucial to the ability of bacteria to penetrate plant roots. Putative complex carbohydrate-degrading enzymes have been found in the analysed genome sequences of three *Azospirillum* species, conferring greater cellulolytic activity^64^. Therefore, these data strongly suggest that the CdgD protein plays a role in bacterial entry into the host plant; however, further research are needed to test this hypothesis.

In addition, under growth in sterile soil conditions, we tested the contribution of the *cdgD* gene to the colonization of the rhizosphere by comparing the ability of the mutant versus the WT and complemented mutant strains to colonize the roots of wheat seedlings. The results indicated less colonization by the mutant compared to the WT and complemented mutant strains, suggesting that unidentified factors, other than motility and biofilm formation, are affected in the 12-A mutant and contribute to the fitness of *A. brasilense* in the rhizosphere colonization (Fig. 7b).

## Conclusions

*Azospirillum brasilense* is a PGPB that has been shown to promote plant growth in several host plants and must therefore successfully colonize the plants, but how colonization is established by the bacterium has not been fully elucidated. Single-gene mutations in predicted related genes encoding DGC and PDE proteins do not result in striking changes in phenotypes that are typically associated with these proteins, such as swimming motility, the production of extracellular polymeric substance components of biofilm matrixes, or host colonization in other bacteria^13–15,38,49,52,63^. Here we show that the inactivation of a gene encoding a hybrid protein that likely behaves as a DGC to decrease c-di-GMP cellular levels functions in motility, production of extracellular polymeric substance components of biofilm matrixes, or plant root surface colonization, indicating its participation in key functions implicated in the interaction of the bacterium with the host plant. Our results also highlights that nutritional status, such as observed here in response to nitrogen availability, has a critical and differential effect on biofilm formation and the production of surface-exposed polymeric substances by *A*. *brasilense* cells. The increase in the diameter of the swimming motility ring is observed in the presence of proline as a carbon source in the parent and mutant strains overexpressing *cdgD* from a plasmid. These data suggest proline may affect CdgD protein function, possibly through its CHASE signaling domain. Motility mediated by the polar flagellum is critical to the performance of the bacterium swimming in soil water, its chemotaxis towards root exudates and its subsequent attachment to the rhizoplane of root plants, thereby maintaining the sustainable growth of its community in the natural environment. After biofilm formation, the surrounding extracellular matrix provides a structure for the maintenance of biofilm cells, keeping them in long-term close proximity; thus, intense interactions occur with such effects as protecting the community from stresses, such as desiccation, contact with biocides and heavy metals, and ultraviolet radiation, which is essential in the competitive habitat of the soil. An mCherry transcriptional reporter fused to the promoter of the *cdgD* gene confirmed that expression of the *cdgD* gene was increased in the stationary phase under abiotic and biotic conditions in vitro and during the association of *A. brasilense* with wheat roots. This result is consistent with the colonization assay, suggesting that CdgD functions to promote an association with plant roots strong interaction that substantially contributes to better adaptation to changing environmental conditions and guarantees the beneficial effects of the bacterium on the plant host.

## Supplementary Information

Supplementary Information 1.

Supplementary Video 1.

Supplementary Video 2.

Supplementary Video 3.

Supplementary Video 4.

## Data Availability

The raw data supporting the conclusions of this article will be made available by the authors, without undue reservation, to any qualified researcher.
