# Supplementary material for: Expression and function of the cdgD gene, encoding a CHASE–PAS-DGC-EAL domain protein, in Azospirillum brasilense
Source: Sci Rep. 2021 Jan 12;11:520. doi: 10.1038/s41598-020-80125-3 (PMC7804937; doi:10.1038/s41598-020-80125-3)
Supplement: Supplementary file 1 — Supplementary Information 1. [file 41598_2020_80125_MOESM1_ESM.pdf]

Supplementary Material

Expression and function of the *cdgD* gene, encoding a CHASE-PAS-DGC-EAL domain protein, in *Azospirillum brasilense*

José F. Cruz-Pérez, Roxana Lara–Oueilhe, Cynthia Marcos-Jiménez, Ricardo Cuatlayotl-Olarte, María L. Xiqui-Vázquez, Sandra R. Reyes-Carmona, Beatriz Eugenia Baca, and Alberto Ramírez-Mata*.

Laboratorio de la interacción bacteria-planta, Centro de investigaciones en Ciencias Microbiológicas, Benemérita Universidad Autónoma de Puebla, Avenida San Claudio S/N, Puebla Pue. México.

Table S1. Strains and plasmids used in this study

Strains	Genotype or phenotype	Reference
Escherichia coli DH5α	F^- *endA*1 *glnV*44 *thi*-1 *recA*1 *relA*1 *gyrA*96 *deoR* *nupG* Φ80*dlacZ*ΔM15 Δ(*lacZYA*-*argF*) U169, *hsdR*17(r_K^- m_K^+), λ^-	[1]
Escherichia coli S17.1	*recA, thi, pro, hsdR*-M + RP4-2-Tc::Mu::Km:Tn7	[2]
Escherichia coli DH5α (p*cdgD*-CI)	Strain containing the plasmid pGEM-T Easy, with the promoter of the *cdgD* gene.	This study
Escherichia coli DH5α FPD-pAZBRmCh	Derivative strain containing the suicide plasmid pFPD-pAZBRmCh, Km^R Tc^R containing the transcriptional fusion (*pcdgD*::*mCherry*)	This study
Escherichia coli S17.1 (pFY4535)	Derivative strain containing the plasmid pFY4535 carrying out the c-di-GMP biosensor, Gm^R	This study
A. brasilense Sp245	Wild-type strain isolated from the wheat roots	[3]
A. brasilense T7mCh	Derivative strain from *A. brasilense* Sp245 strain with a chromosomic *mCherry* gene without a promoter, Km^R.	This study
A. brasilense 12-A	Derivative strain from *A. brasilense* Sp245 strain mutated in the *cdgD* gene, Km^R.	This study
A. brasilense C-56A	Derivative strain from *A. brasilense* 12-A containing the plasmid pVK*cdgD*, Km^R, Tc^R.	This study
A. brasilense C-40A	Mutant 12-A containing the empty vector pVK100, Km^R, Tc^R	This study
A. brasilense FPDm1	Derivative from Sp245 strain with the chromosomic transcriptional fusion *pcdgD-mCherry*, Km^R.	This study
A. brasilense Sp245 (pMP2449-5)	Derivative from Sp245 strain, containing the pMP2449-5 plasmid which carries out *mCherry* gene, Gm^R	[4]
A. brasilense 12-A (pMP2449-5)	Derivative from *A. brasilense* 12-A strain, containing the pMP2449-5 plasmid which carries out *mCherry* gene, Gm^R	This study
A.brasilense C-56A (pMP2449-5)	Derivative from *A. brasilense* C-56A strain, containing the pMP2449-5 plasmid which carries out *mCherry* gene, Gm^R	This study

Strains	Genotype or phenotype	Reference
A.brasilense C-40A (pMP2449-5)	Derivative from *A. brasilense* C-40A strain, containing the pMP2449-5 plasmid which carries out *mCherry* gene, Gm^R	This study
A. brasilense Sp245 (pFY4535)	Derivative from Sp245 strain, containing the pFY4535 plasmid, Gm^R	This study
A. brasilense 12-A (pFY4535)	Derivative from 12-A strain, containing the plasmid pFY4535, Gm^R	This study
A. brasilense C-56A (pFY4535)	Derivative from C-56A strain, containing the plasmid pFY4535, Gm^R	This study
A. brasilense C-40A (pFY4535)	Derivative from C-40A strain, containing the plasmid pFY4535, Gm^R	This study
**Plasmids**	**Genotype or phenotype**	**Reference**
pGEM-T-Easy	Cloning vector *ori* f1, *lacZa* Ap^R	Promega
pCR 2.1 TOPO	Cloning vector *ori* pUC, *ori* f1, *lacZa* Ap^R Km^R	Thermo Fisher Scientific
pVK100	Broad host range cloning cosmid vector, *cos* site of λ phage, Km^R, Tc^R	[5]
pSUP202	Suicide plasmid, pMB1 replicon, mob^+ Ap^R, Cm^R, Tc^R.	[2]
pBSL98	Vector pBSL carrying the cassette Km^R	[6]
pJMS-Km^R	Suicide plasmid derivative from pSUP202, mob^+ Ap^R, Tc^R, Km^R .	[7]
pFY4535	Derived from pMMB67EH (Gm) containing the c-di-GMP biosensor, with the *hok/sok* region from pXB300, Gm^R	[8]
pCR*cdgD*	Plasmid derivative of pCR 2.1 TOPO carrying out the *cdgD* gene Ap^R Km^R	This study
pAB*cdgD*	Plasmid derivative of pSUP202 carrying out the *cdgD* gene Ap^R, Tc^R.	This study
pAB*cdgD*::Km^R	Construct derivative of pAB*cdgD* carrying out the *cdgD*::Km^R, Ap^R, Tc^R, Km^R.	This study
pVK*pcdgD*	Construct derivative of pVK100 contains the *pcdgD* gene with its native promoter, Tc^R, Km^R.	This study
pAZBR-T7mCh	Construct derivative of pJMS-Km^R, suicide plasmid carrying the chromosomal CCC96879.1 and CCC96880.1 putative proteins from *A. brasilense* Sp245 strain, a MSC, *mCherry* reporter gene, ΩT7 terminator. Km^R, Tc^R.	This study
pGEM-*pcdgD*	Construct derivative of pGEM-TEasy containing a fragment of 456 bp harbor the regulatory region of the *cdgD* gene. Ap^R.	This study

Strains	Genotype or phenotype	Reference
pAB79	Construct derivative of pGEM-TEasy containing a 5'fragment of 700 bp carrying the chromosomal CCC96879.1 gene from *A. brasilense*	This study
pAB80	Construct derivative of pGEM-TEasy containing a 3'fragment of 900 bp carrying the chromosomal CCC96880.1 gene from *A. brasilense*	This study
pAZBR-*pcdgD*T7mCh	Construct of derivative of pAZBR-T7mCh suicide plasmid carrying out the fragment of 456 bp (regulatory region of the *cdgD*) inserted in 5 ´ *mCherry* reported gene, Km^R, Tc^R	This study
pMP2449-5	pMP2449-5 plasmid which carries out *mCherry* gene, Gm^R	[4]

Resistance: Ampicillin =Ap^R; Gentamycin =Gm^R; Kanamycin =Km^R; Tetracycline =Tc^R.

Table S2. Primers designed and used in this study

Notation	Forward primer (5´- 3´)	Reverse primer (5´- 3´)	Reference
M13F/ M13 R	GTAAAACGACGGCCAGT	GTCATAGCTGTTTCCTG	Thermo Fisher Scientific
glyAF/ glyAR	GGAGATCGCCAAGAAGATCA	GCTCTTGGCGTAGGTCTTGA	[9]
MTF-*SpeI*F / MTF-*Nco*IR	CG**ACTAGT**CGCTCTGGTTTAT TGGAGCTTT	TT**CCATGG**ATGACCACGCCCGAC AGCA	This study
CMP-*PstI*F/ CMP-*Kpn*IR	GG**CTGCAG**TTGGACGCCCATTC GGCCC	CC**GGTACC**GGACGGCGTTCACCC GATCA	This study
TraJ/OriT	TCTTCTTGATGGAGCGCATGG	CTGCTTCGGGGTCATTATAGC	This study
P-*cdg*D-F/(*Sna*B1) P-*cdgD*-R/ (*Xho*I)	A**TACGTA**GCGGGGGACGAACT GCCGTCC	A**CTCGAG**CCCATGCCGCCCAC CGCC	This study
T7iorA	AGGTACCCTAGCATAACCCCTT GGGGCCTCTAAACGGGTCTTGA GGGGTTTTTTGCGGCCGCTACGT ACCTGCGCTGGACCATCAGCA	-	This study
qRT-*cdgD*F/ qRT-*cdgD*R	CCAGATCCAGGTCTATGTTCAG	GTTCCATTCCGTGCGTTTC	This study
4571RC-F 4571DC-R	GTGGCGGGGCGATGGGGAAGG	GCGGCTGGGTGCGGGACAAC	This study
F-ORF210 (*Hind*III) R-ORF-210 (*Xho*I)	AGA**AAGCTT**AGATCCTGGGCA TGATCGAG	AGC**CTCGAG**AGAAGGCGTGGT GACCTTTC	This study

The restriction sequences are in bold.

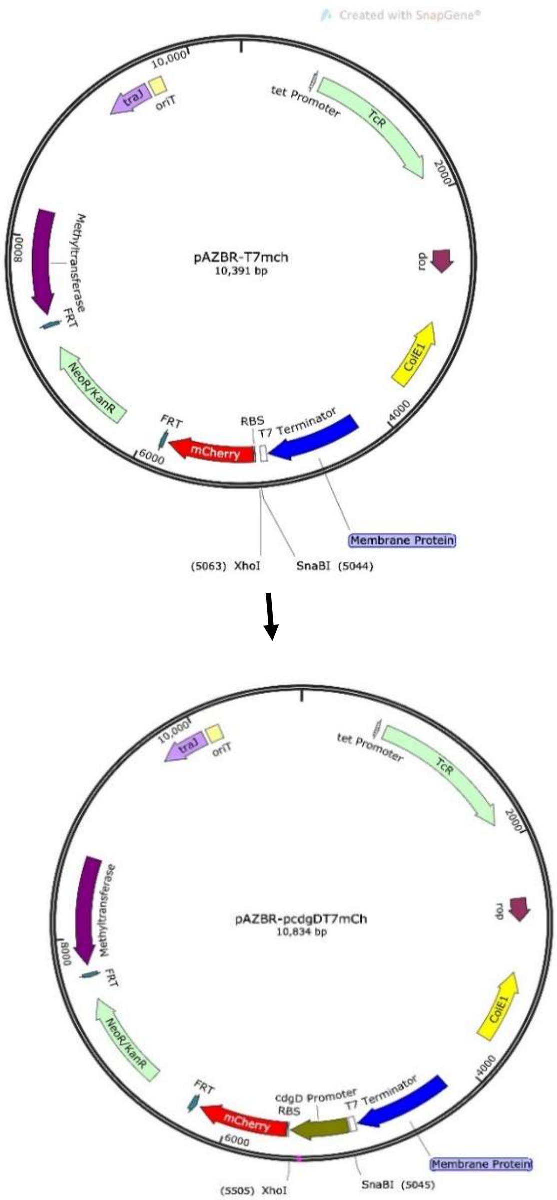

Figure S1. Plasmid maps constructed and used in this study

The plasmid pAZBR-T7mCh was constructed from the pJMS-Km^R suicide vector [7], as follows: The CMP-*Pst*IF/CMP-*Kpn*IR primers (Table S2) were employed to amplify a 5´ fragment measuring 700 bp that included the ORF (GenBank accession number CCC96879.1, encoding a membrane protein of unknown function), which was then cloned into pGEM-T-Easy (Promega, Madison, WI, USA) to generate the pAB79 plasmid. Subsequently, primers MTF-*Spe*IF/MTF-*Nco*IR (Table S2) were used to amplify a 3´ fragment measuring 900 bp including the ORF (GenBank accession number CCC96880.1, encoding a putative SAM-dependent methyltransferase), which was further cloned into pGEM-T-Easy to

yield the pAB80 plasmid. Both fragments were excised from plasmids with the corresponding restriction enzymes and cloned into pJMS-Km^R to obtain a suicide plasmid containing the mCherry gene without a promoter. Subsequently, the terminator T7 sequence (named T7iorA, Table S2) was introduced between the *mCherry* and CCC96879.1 genes to guarantee transcription termination. This plasmid was designated pAZBR-T7mCh. This plasmid was modified with an insertion into the 5′ region of the *mCherry* gene of a fragment 456 bp upstream of the *cdgD* gene including its promoter, which had been previously amplified with the primers P-*cdgD*-R/(*Xho*I) and P-*cdgD*-F/(*Sna*BI) (Table S2) and cloned into pGEM-T-Easy to yield the pGEM-*pcdg*D plasmid. Both the pAZBR-T7mCh and pGEM-*pcdg*D plasmids were digested with *Xho*I and *Sna*B1. Then, cloning was performed to obtain the pAZBR-*pcdgD*T7mCh plasmid, which was verified by PCR (using the primers M13F/M13R and TraJ/OriT) and subsequently sequenced (Table S2).

a

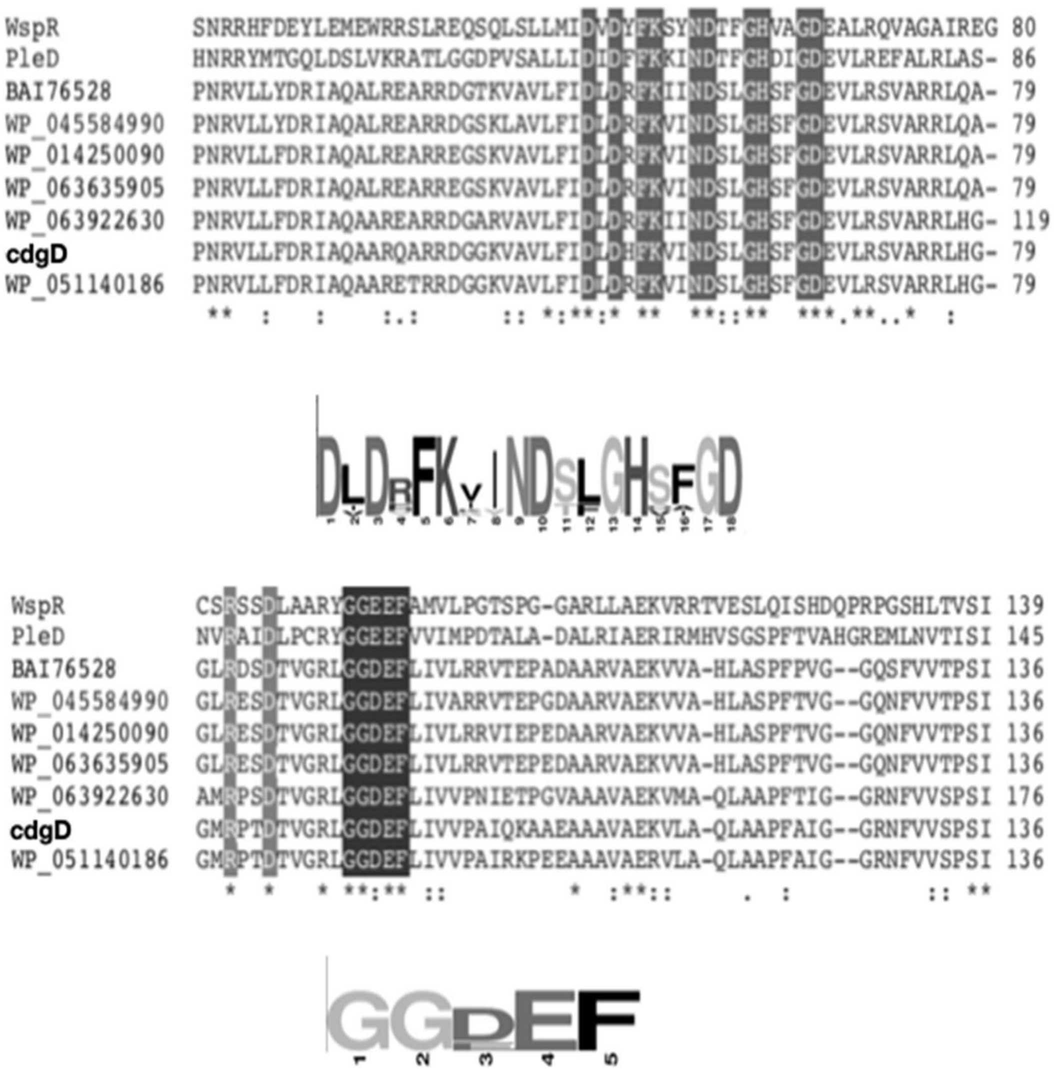

Figure S2a. Alignment of sequences, depicting the amino acid residues of the GGDEF domain

Alignments were created and analysed by Clustal-omega to compare the motifs. The protein product of the *cdgD* gene was compared with the WspR protein of *Pseudomonas aeruginosa* or PleD of *Caulobacter crescentus*, while the diguanylate cyclase (DGC) protein served as a reference for the amino acid motifs of the GGDEF protein domain (https://www.ebi.ac.uk/Tools/msa/clustalo/). WspR, *Pseudomonas aeruginosa*; PleD, *Caulobacter crescentus; Azospirillum* sp. B510 (BAI76528)*;* *A. thiophilum* BV-S (WP_045584990); *A. lipoferum* 4B (WP_014250090); *A. humicireducens* SgZ-5 (WP_063635905); *A. brasilense* Az39 (WP_063922630); *cdgD*, *A. brasilense* Sp245, *A. brasilense* Sp7 (WP_051140186). The conserved motifs are shaded, and conserved amino acid residues are indicated with asterisks.

b

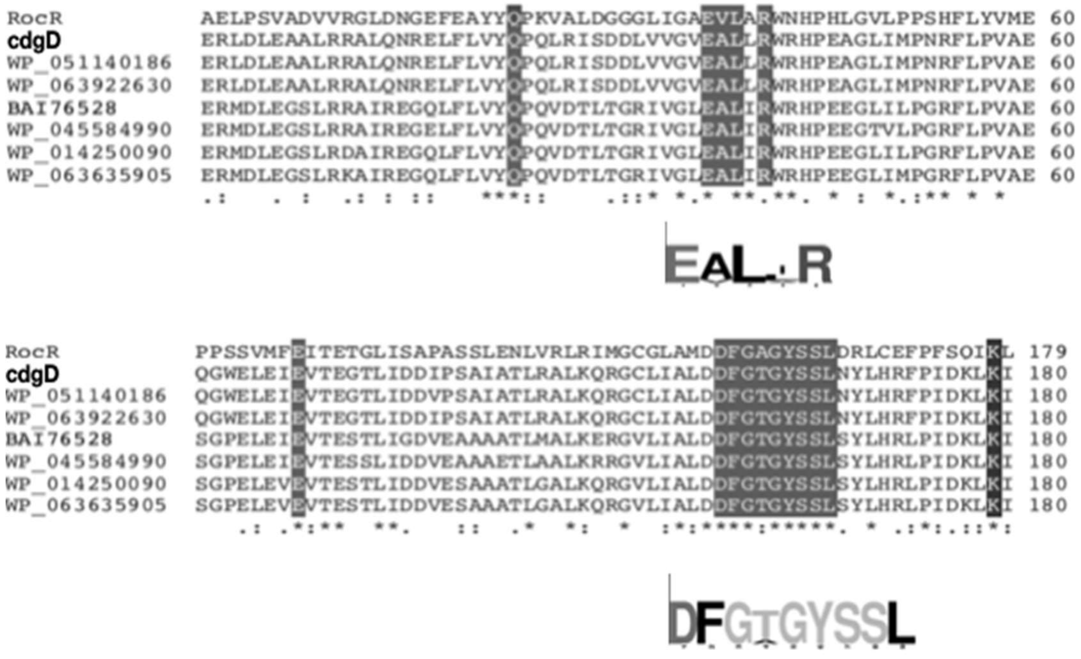

Figure S2b. Alignment of sequences, depicting the amino acid residues of the EAL domain

Alignments were created and analysed by Clustal-omega to compare the motifs of the protein product of the *cdgD* gene with those of the RocR protein of *Pseudomonas aeruginosa*, while phosphodiesterase protein served as a reference for the amino acid motifs of the EAL protein domain (https://www.ebi.ac.uk/Tools/msa/clustalo/). RocR *Pseudomonas aeruginosa*; *cdgD A. brasilense* sp245*; A. brasilense* Sp7 (WP_051140186); *A. brasilense* Az39~~,~~ (WP_063922630); *Azospirillum* sp. B510 (BAI76528); *A. thiophilum* BV-S, (WP_045584990); *A. lipoferum* 4B~~,~~ (WP_014250090); *A. humicireducens* SgZ-5~~,~~ (WP_063635905). The conserved motifs are shaded, and conserved amino acid residues are indicated with asterisks.

Homology modelling and *in silico* analysis

The CdgD protein sequence from *A. brasilense* Sp245 was retrieved from the NCBI database (https://www.ncbi.nlm.nih.gov/protein; Accession number; AZOBR_100210). The Rapid Annotation using Subsystem Technology (RAST) server was employed for the initial analysis. Protein domain predictions were obtained using SMART[10] (http://smart.embl-heidelberg.de), HMMER[11] and PROSITE[12]. To predict and identify transmembrane regions, the TMHMM[13] and TMpred tools were used[14] (Table S3 and Figures S3 and S4). The visualization of the transmembrane helices was performed with the HeliQuest helical wheel-drawing program[15], Figure S4. Secondary structure predictions were performed using the Phyre2 and I-Tasser programs to detect the domain organization and to identify a suitable template fold for CHASE and PAS-GGDEF-EAL domains[16]. Three-dimensional models of the CHASE (residues 64-327) and PAS-GGDEF-EAL (residues 378-946) were obtained using the Phyre2 and I-Tasser packages[16, 17] and analysed by Chimera software[18] using the following crystal structure as structural templates: the CHASE domain of the PcrK a histidine kinase of *Xanthomonas campestris* pathovar *campestris* (PDB Code: 6K62)[19], the PAS-GGDEF-EAL domains of the RbdA protein from *Pseudomonas aeruginosa* (PDB Code: 5XGB)[20].

Table S3. Summary of Phyre2 and I-Tasser servers from CdgD protein

Region (aa)	Fold	Best Templates (PDB code)	Phyre2 Confidence	I-TASSER TM-Score	I-TASSER RMSD (Å)
46-63	TM1	-	-	-	-
64-327	CHASE	6K62	100%	0.928	0.74
338-361	TM2	-	-	-	-
378-489	PAS	5XGB	100%	0.918	0.79
534-669	GGDEF	5XGB	100%	0.918	0.79
690-920	EAL	5XGB	100%	0.918	0.79

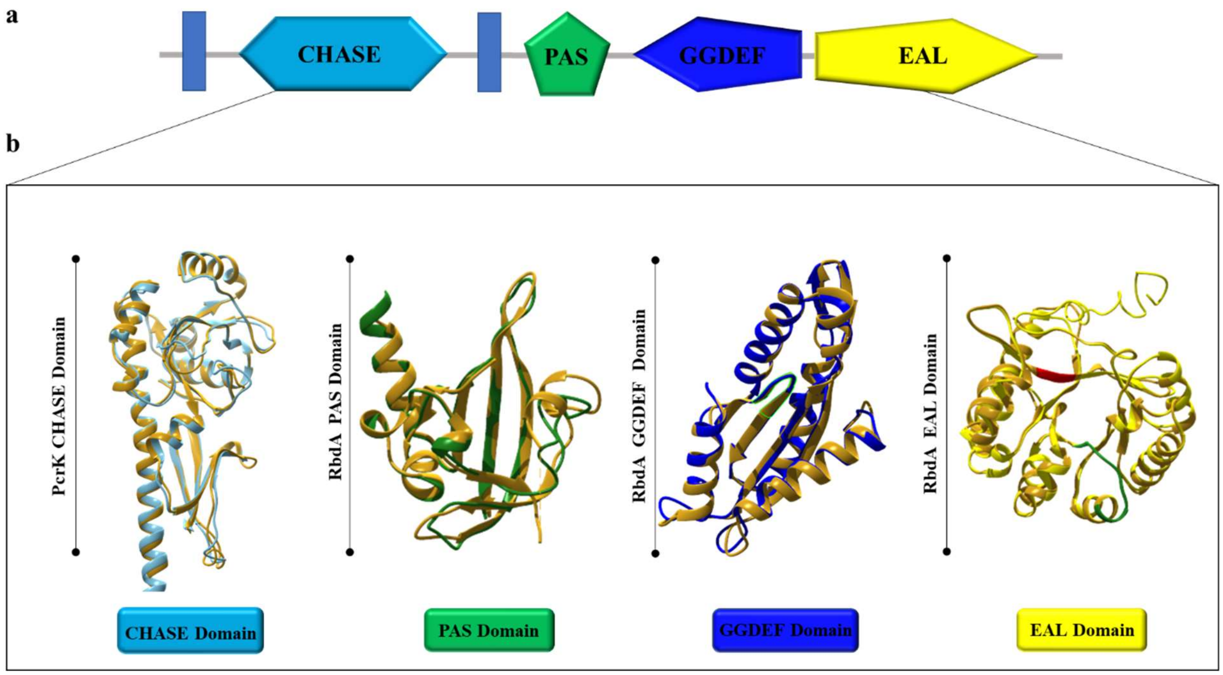

Figure S3. *In silico* analysis of the CdgD protein domains (**a**) Domain organization of CdgD along with its primary structure, (**b**) Structure superposition of each CdgD domain (gold) with the other known structures. The PcrK CHASE domain of *Xanthomonas campestris* pathovar *campestris* (light blue), the cRbdA PAS, and GGDEF domains from *Pseudomonas aeruginosa* (green and dark blue, respectively), and the cRbdA EAL domain from *Pseudomonas aeruginosa* (yellow).

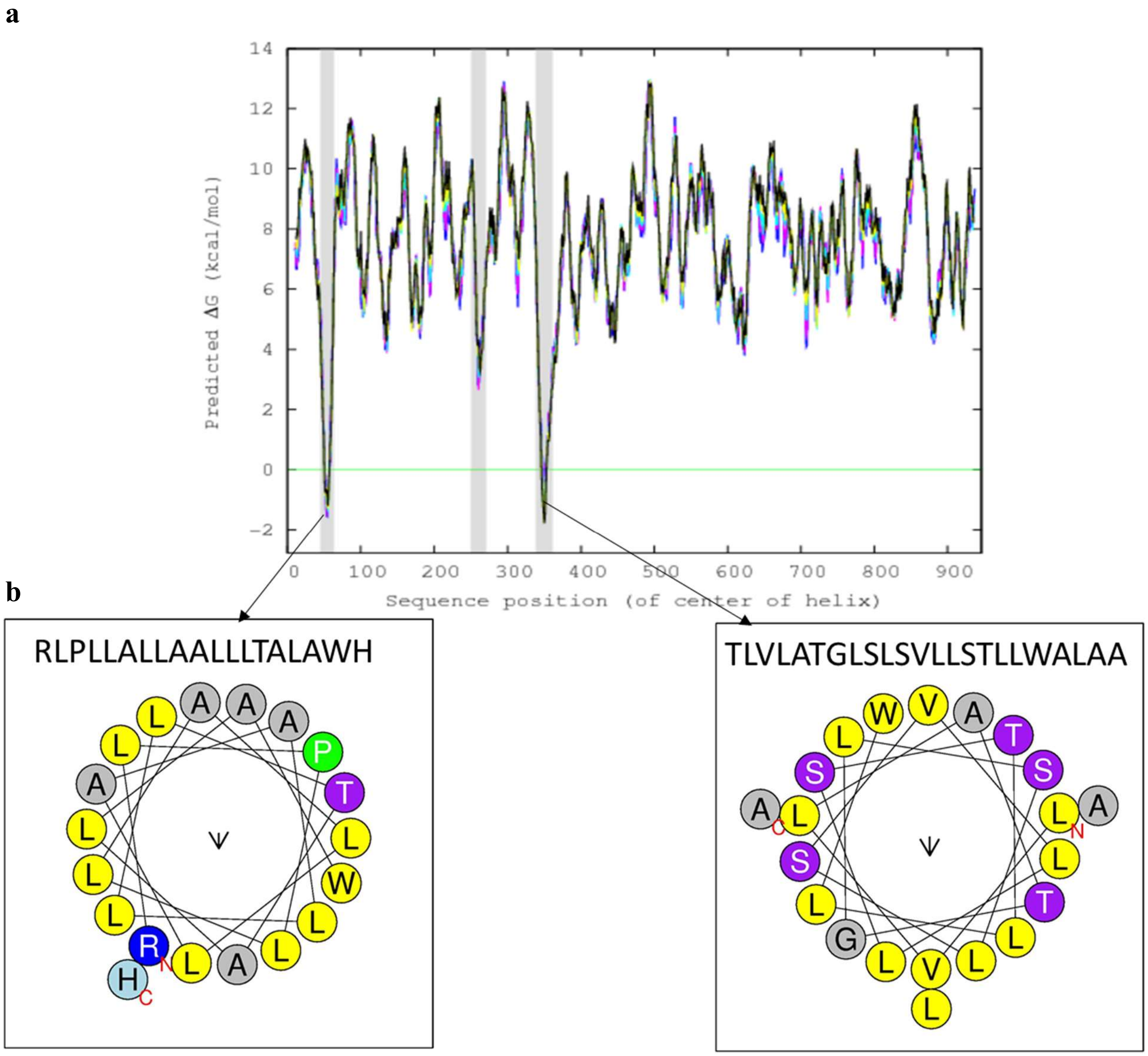

Figure S4. Analysis of the transmembrane helices of CdgD. (a) Detection of putative transmembrane helices at the N-terminal end. The probability that a residue belonged to a transmembrane helix was calculated for the first 380 amino acids of CdgD with a Markov model by the TMHMM server. (**b)** Projection of the helical wheel was made using HeliQuest software (http://heliquest.ipmc.cnrs.fr/cgi-bin/ComputParams.py).

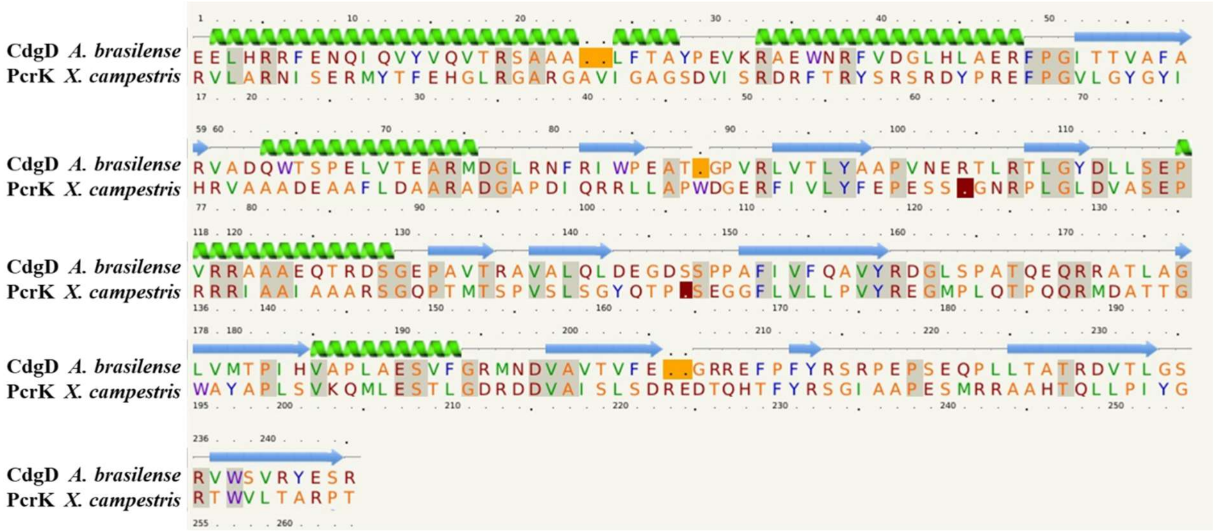

Figure S5. Sequence comparison of the CHASE domain of CdgD of *A. brasilense* Sp245 and the two-component system sensor histidine kinase PcrK of *Xanthomonas campestris* pv *campestris*. CdgD (accession number AZOBR_100210) and PcrK (accession number WP_011037503). Secondary structure elements of the CHASE domain are displayed above and below the alignment.

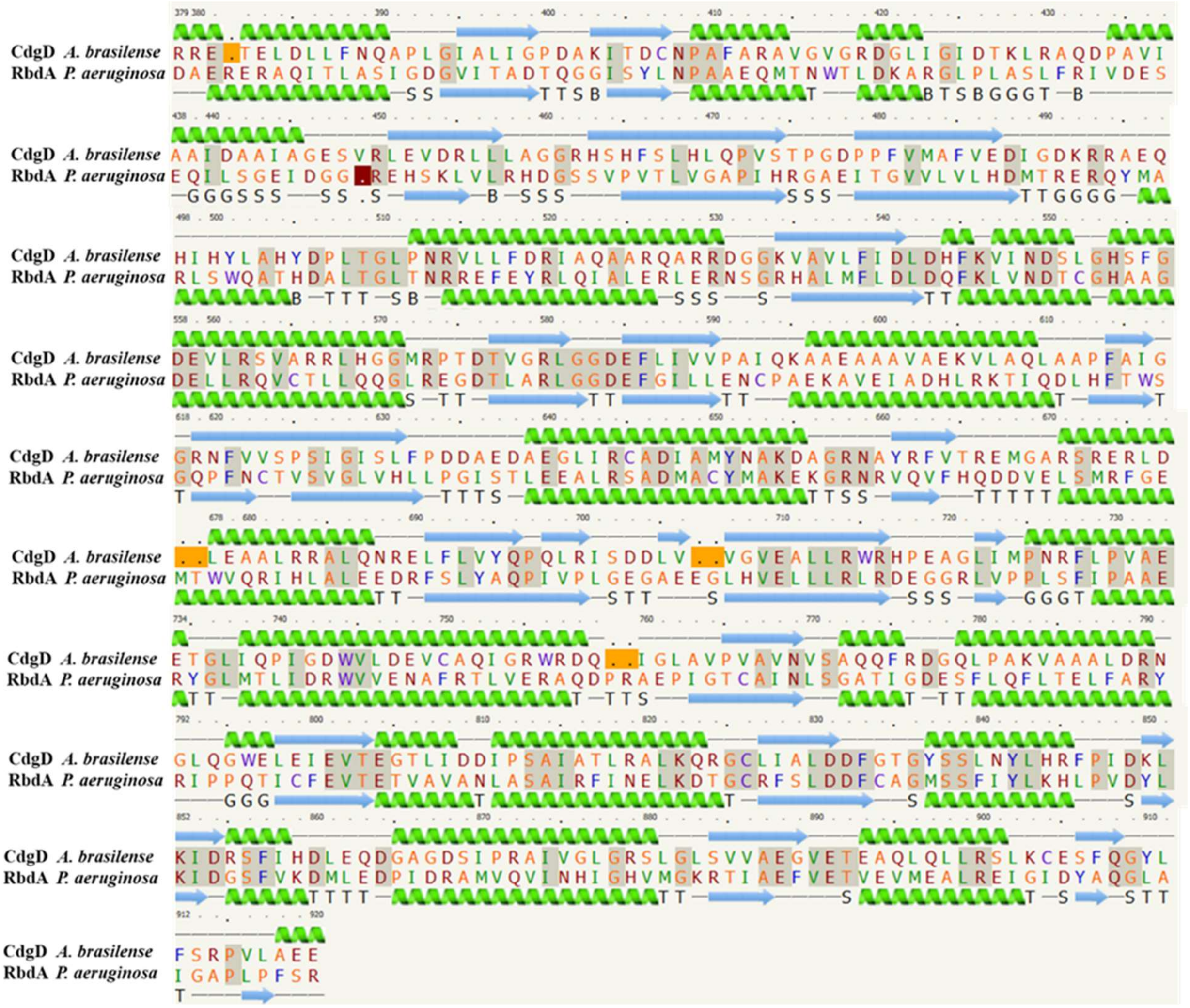

Figure S6. Sequence comparison of the PAS, GGDEF, and EAL domains of CdgD and RbdA. CdgD of *A. brasilense* Sp245 (accession number AZOBR_100210) and RbdA of *Pseudomonas aeruginosa* (accession number Q91580) are depicted. Secondary structure elements of the PAS-GGDEF-EAL domains of CdgD and RbdA are displayed above and below the alignment.

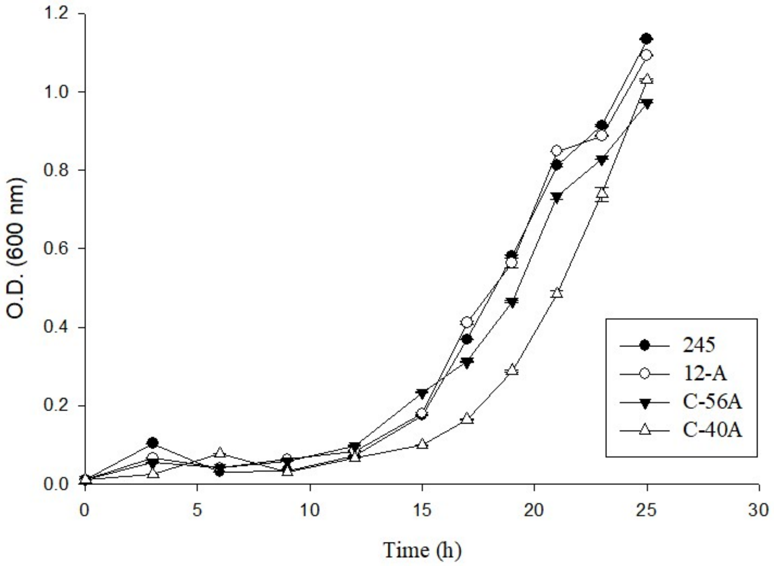

Figure S7. **Growth curves of *A. brasilense* Sp245, *A. brasilense* 12-A, *A. brasilense* C-56A, and *A. brasilense* C-40A.** The strains were grown in NFB* medium, with KNO_3 as a nitrogen source. Cultures were kept for 25 h at 30°C under agitation (120 rpm). Optical density was performed at 600 nm. Values are means ± standard error of three independent cultures.

Time-lapse images of *A. brasilense* strains under free-swimming conditions.

The *A. brasilense* strains 245, 12-A, C-40A, and C-56A were grown in LB* liquid medium overnight with shaking at 30°C. Next, these cultures were diluted to 1:100 in fresh NFB*+KNO3 broth supplemented with 10 mM proline and incubated for 24 h at 30°C with shaking at 120 rpm. Each *Azospirillum* strain was visualized under a 100x objective (oil Ph3 DL lens) using an inverted microscope (Nikon TE2000U), and videos of bacteria were acquired at 15 frames per second using a camera (Nikon DS-Qi1Mc) controlled by NIS-Elements BR 4.20 software. The gain (9.6x) and the exposure (50 ms) settings were maintained at the same level during the capture of every image set.

Movie S1. Video shows wild-type *Azospirillum brasilense* Sp245 cells under free-swimming conditions.
Movie S2. Video shows *Azospirillum brasilense* 12-A cells under free-swimming conditions.
Movie S3. Video shows *Azospirillum brasilense* C-56A cells under free-swimming conditions.
Movie S4. Video shows *Azospirillum brasilense* C-40A cells under free-swimming conditions.

References

1 Hanahan, D. Studies on transformation of Escherichia coli with plasmids. *Journal of molecular biology* **166**, 557-580, doi:10.1016/s0022-2836(83)80284-8 (1983).

2 Simon, R., Priefer, U. & Pühler, A. A Broad Host Range Mobilization System for In Vivo Genetic Engineering: Transposon Mutagenesis in Gram Negative Bacteria. *Bio/Technology* **1**, 784-791, doi:10.1038/nbt1183-784 (1983).

3 Baldani, V. L. D., Baldani, J. I. & Döbereiner, J. Effects of Azospirillum inoculation on root infection and nitrogen incorporation in wheat. *Canadian Journal of Microbiology* **29**, 924-929, doi:10.1139/m83-148 (1983).

4 Ramirez-Mata, A., Pacheco, M. R., Moreno, S. J., Xiqui-Vazquez, M. L. & Baca, B. E. Versatile use of Azospirillum brasilense strains tagged with egfp and mCherry genes for the visualization of biofilms associated with wheat roots. *Microbiological research* **215**, 155-163, doi:10.1016/j.micres.2018.07.007 (2018).

5 Knauf, V. C. & Nester, E. W. Wide host range cloning vectors: a cosmid clone bank of an Agrobacterium Ti plasmid. *Plasmid* **8**, 45-54, doi:10.1016/0147-619x(82)90040-3 (1982).

6 Alexeyev, M. F., Shokolenko, I. N. & Croughan, T. P. Improved antibiotic-resistance gene cassettes and omega elements for Escherichia coli vector construction and in vitro deletion/insertion mutagenesis. *Gene* **160**, 63-67, doi:10.1016/0378-1119(95)00108-i (1995).

7 Ramírez-Mata, A. *et al.* The cyclic-di-GMP diguanylate cyclase CdgA has a role in biofilm formation and exopolysaccharide production in Azospirillum brasilense. *Research in Microbiology* **167**, 190-201, doi:https://doi.org/10.1016/j.resmic.2015.12.004 (2016).

8 Zamorano-Sanchez, D. *et al.* Functional Specialization in Vibrio cholerae Diguanylate Cyclases: Distinct Modes of Motility Suppression and c-di-GMP Production. *mBio* **10**, doi:10.1128/mBio.00670-19 (2019).
9 McMillan, M. & Pereg, L. Evaluation of Reference Genes for Gene Expression Analysis Using Quantitative RT-PCR in Azospirillum brasilense. *PloS one* **9**, e98162, doi:10.1371/journal.pone.0098162 (2014).
10 Letunic, I., Doerks, T. & Bork, P. SMART: recent updates, new developments and status in 2015. *Nucleic acids research* **43**, D257-260, doi:10.1093/nar/gku949 (2015).
11 Finn, R. D. *et al.* HMMER web server: 2015 update. *Nucleic acids research* **43**, W30-38, doi:10.1093/nar/gkv397 (2015).
12 de Castro, E. *et al.* ScanProsite: detection of PROSITE signature matches and ProRule-associated functional and structural residues in proteins. *Nucleic acids research* **34**, W362-365, doi:10.1093/nar/gkl124 (2006).
13 Krogh, A., Larsson, B., von Heijne, G. & Sonnhammer, E. L. Predicting transmembrane protein topology with a hidden Markov model: application to complete genomes. *Journal of molecular biology* **305**, 567-580, doi:10.1006/jmbi.2000.4315 (2001).
14 Hofmann, K. TMBASE-A database of membrane spanning protein segments. *Biol. Chem. Hoppe-Seyler* **374**, 166 (1993).
15 Gautier, R., Douguet, D., Antonny, B. & Drin, G. HELIQUEST: a web server to screen sequences with specific alpha-helical properties. *Bioinformatics* **24**, 2101-2102, doi:10.1093/bioinformatics/btn392 (2008).
16 Yang, J. *et al.* The I-TASSER Suite: protein structure and function prediction. *Nature methods* **12**, 7-8, doi:10.1038/nmeth.3213 (2015).
17 Kelley, L. A., Mezulis, S., Yates, C. M., Wass, M. N. & Sternberg, M. J. The Phyre2 web portal for protein modeling, prediction and analysis. *Nature protocols* **10**, 845-858, doi:10.1038/nprot.2015.053 (2015).
18 Pettersen, E. F. *et al.* UCSF Chimera--a visualization system for exploratory research and analysis. *Journal of computational chemistry* **25**, 1605-1612, doi:10.1002/jcc.20084 (2004).
19 Chen, P. *et al.* The crystal structure of the phytopathogenic bacterial sensor PcrK reveals different cytokinin recognition mechanism from the plant sensor AHK4. *Journal of structural biology* **208**, 69-76, doi:10.1016/j.jsb.2019.08.001 (2019).
20 Liu, C. *et al.* Insights into Biofilm Dispersal Regulation from the Crystal Structure of the PAS-GGDEF-EAL Region of RbdA from Pseudomonas aeruginosa. LID - 10.1128/JB.00515-17 [doi] LID - e00515-17. *J. Bacteriol* (2018).
